# Primate retroelement exonization and sexually dimorphic *IL13RA1* transcription tune type 2 immune responses

**DOI:** 10.1126/sciimmunol.adr1105

**Published:** 2025-07-04

**Authors:** Tobias Plowman, Tom Hofland, Callum Hall, Rachael Thompson, Judith Pape, Kevin W Ng, Laura Doglio, George Kassiotis

**Affiliations:** 1 Retroviral Immunology Laboratory, The Francis Crick Institute; London, UK; 2 Department of Infectious Disease, Faculty of Medicine, Imperial College London; London, UK

## Abstract

Type 2 immunity is orchestrated by IL-4 and IL-13 signaling, initiated by binding to receptors that are specific to each cytokine or to the shared heterodimeric receptor comprising the IL-4Rα and IL-13Rα1 subunits. Here, we report that sexually dimorphic *IL13RA1* transcription, is regulated by estrogen, and characterize an IL-13Rα1 isoform (referred to here as IL-13Rα1-LOR1a), created through facultative splicing to an alternative terminal exon composed of primate-specific retrotransposable elements (RTEs). At the mRNA level, RTE exonization replaces regulatory sequences in the canonical 3′ untranslated region (UTR) implicated in *IL13RA1* mRNA stability. Moreover, alternative splicing removes critical domains in the cytoplasmic tail, rendering the IL-13Rα1-LOR1a isoform partially signaling-defective at the protein level. When coexpressed, the IL-13Rα1-LOR1a isoform antagonizes the function of the canonical receptor, reducing cellular responsiveness to IL-4 and IL-13. Thus, the balance of the two *IL13RA1* isoforms appears to fine-tune type 2 cytokine signaling and downstream immune responses.

## Introduction

Type 2 immunity contributes to host protection against infection or parasitization, but its dysregulation may cause fibrotic or allergic diseases (1–4). The induction and regulation of type 2 immunity are orchestrated by type 2 cytokines, including the prototypical interleukin (IL)-4 and IL-13, which drive the differentiation of CD4 T cells into T helper (T_H_)2 cells, alternative activation of macrophages, and IgG1 and IgE class-switching by B cells, among other effects (1–4).

IL-4 and IL-13 can signal through the shared type II receptor, a heterodimer composed of the IL-4Rα and IL-13Rα1 subunits, which is expressed by all cell types except T cells (2, 5). IL-4 can also bind the type I receptor, a heterodimer of IL-4Rα and the IL-2Rγ (common γ) chain, a combination expressed only by hematopoietic cells (2, 5). Consequently, IL-4, IL-13, and their shared receptors exert overlapping, as well as non-redundant biological activities (2, 5). Indeed, reduced basal IgE levels have been reported in mice deficient in IL-13 (6) or in IL-13Rα1 (7), whereas T cell–dependent IgE production in response to parasitism or allergen challenge is variably affected by IL-13Rα1 deficiency (7–9), indicating compensatory mechanisms.

Polymorphisms in the human *IL13RA1* locus on chromosome Xq24 (encoding IL-13Rα1), particularly in interaction with *IL13* polymorphisms, have been linked with serum IgE levels in childhood and in utero (10–13). IgE sensitization, the production of allergen-specific IgE, is an integral component of allergic disorders, which develop most frequently in early childhood, with a higher prevalence in males, and in groups sharing genetic ancestry or associated environmental exposure (14–19). Genetic associations among *IL13RA1* and *IL13*, biological sex, and other genetic attributes indicate an essential, but as yet incompletely understood role for type 2 cytokine signaling in IgE sensitization.

Retrotransposable elements (RTEs) are a source of prior unknown genetic influence on gene expression (20–22). Alternative transcript and encoded protein isoforms can be generated through exonization of ancient retroviral or retroviral-like integrations, providing a source of evolutionary diversity in the function of host processes such as immunity (20–22). Previous examples highlighted the potential of RTEs to modify biological outcome and include the creation of novel isoforms of immune receptors, such as CD5 (23), PD-L1 (24), and IFN-αR2 (25).

Here, we report sexually dimorphic expression of *IL13RA1* and the characterization of a partially signaling-defective IL-13Rα1 isoform created by exonization of a primate-specific RTE that regulates IL-4 and IL-13 responsiveness.

## Results

### *IL13RA1* isoforms created by retroelement exonization

Given the documented differences in IgE sensitization between males and females (14, 15, 18), we asked if *IL13RA1* plays a role in this phenomenon. In data from the Genotype-Tissue Expression (GTEx) portal (26)—a tissue-level RNA sequencing (RNA-seq) data resource—*IL13RA1* was widely but similarly expressed between males and females (fig. S1A). This was also the case for *TLR7* (fig. S1A), a gene with reported sexually dimorphic expression (27–29) and a negative regulator of IgE responses (30, 31) and asthma (32). However, it is in B cells that IL-13Rα1 signaling promotes germline epsilon transcript *IGHE* transcription and subsequent IgE class-switch recombination and production, a prerequisite for IgE sensitization (33). We therefore analyzed a lymphoblastoid B cell line (LCL) dataset (34) and found significantly higher *IL13RA1* expression in males compared to females (fig. S1B). This association was true only for individuals of European ancestry and not for those of Asian or African ancestry (fig. S1B), consistent with reported influences of genetic ancestry on allergy and asthma susceptibility (14–19). By contrast, *TLR7* expression was lower in male LCLs compared to female LCLs from donors of European ancestry (fig. S1B). To confirm our findings, as well as probe potential links with dysregulated type 2 immunity, we analyzed LCLs from children of British ancestry from families with an asthmatic proband (35). *IL13RA1* expression was significantly lower in LCLs from male than from female non-asthmatic siblings (fig. S1C), as might be expected for an X-linked gene with evidence for inactivation escape. However, this pattern was reversed in asthmatic probands, where male LCLs exhibited higher *IL13RA1* expression than female LCLs, whereas *TLR7* expression was comparable (fig. S1C).

**Fig. 1:**
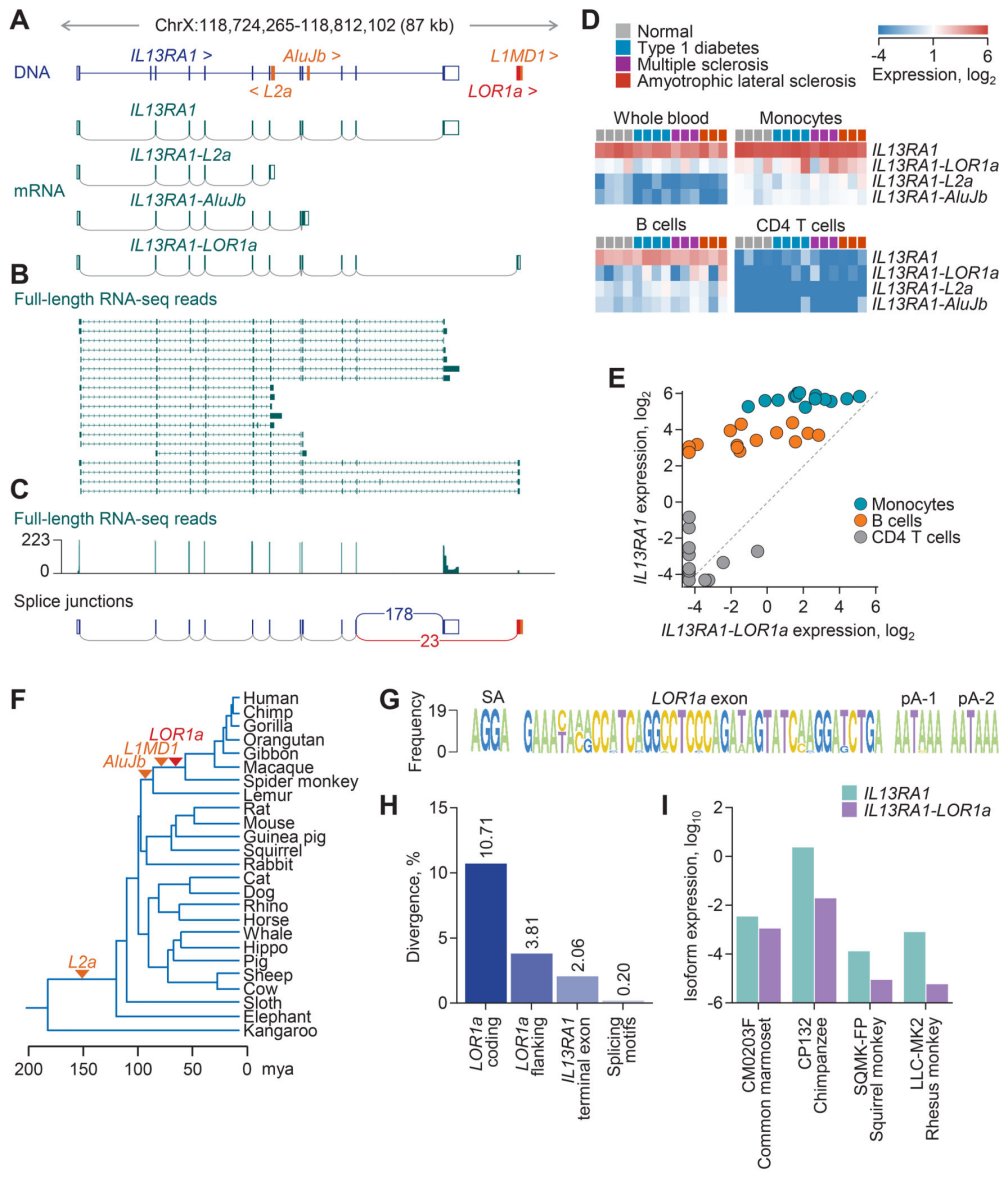
*IL13RA1* isoforms created by retroelement exonization. (**A**) Annotated genomic structure of the *IL13RA1* locus also depicting the relevant RTEs (DNA) and assembled transcripts (mRNA). (**B**) Collapsed transcripts built from full-length RNA-seq reads from healthy human PBMCs (PacBio Multiplexed Arrays Sequencing) in the *IL13RA1* locus. (**C**) Trace of full-length RNA-seq reads (top) and analysis of splice junctions between the penultimate exon and the two alternative terminal *IL13RA1* exons (bottom) in RNA-seq data from healthy human PBMCs (SRR9944890). (**D**) Expression of *IL13RA1* isoforms in RNA-seq data from the indicated peripheral blood cell populations (SRP045500). Columns represent individual healthy donors or patients with the indicated condition (*n* = 14). (**E**) Correlation between *IL13RA1* and *IL13RA1-LOR1a* expression in RNA-seq data from the same samples as in (D). (**F**) Estimation of the evolutionary age of the RTE insertions creating *IL13RA1* isoforms, based on genomic sequence alignments. (**G**) Logo plots for the splice acceptor (SA) site, the *LOR1a* exon and polyadenylation (pA) signals, based on sequence conservation in 19 primate genomes. (**H**) Sequence divergence of the *IL13RA1-LOR1a* coding and splicing motifs in the 19 primate genomes. The canonical *IL13RA1* terminal exon and *LOR1a* flanking sequences are also included for comparison. (**I**) Expression of *IL13RA1* and *IL13RA1-LOR1a*, determined by RT-qPCR and plotted relative to *HPRT1* expression, in cell lines from the indicated primate species (single measurements, *N* = 1).

To further understand its functional relevance in type 2 immunity, we next quantified the expression of *IL13RA1*, distinguishing between mRNA isoforms which may or may not encode a functional protein. Inspection of a prior transcriptome assembly (36) identified three alternative *IL13RA1* isoforms, each created by co-option of distinct germline RTEs integrated near or within the locus (Fig. 1A). Use of an *L2a* element in intron 6 as an alternative terminal exon produces a truncated transcript, referred to here as *IL13RA1-L2a*, matching annotated transcript ENST00000371642.1 (Fig. 1A). Similarly, exonization of an intronic *AluJb* element in intron 8 produces an unannotated truncated transcript, referred to here as *IL13RA1-AluJb* (Fig. 1A). Finally, an alternative transcript, referred to here as *IL13RA1-LOR1a* and matching RefSeq predicted transcript XM_054327031.1, is produced by omission of the canonical terminal exon and use of downstream *LOR1a* and *L1MD1* elements (Fig. 1A). All three RTE-exonizing isoforms were supported by inspection of full-length RNA-seq data from healthy donor peripheral blood mononuclear cells (PBMCs) (37) (Fig. 1B). Splice junction analysis of a separate full-length RNA-seq dataset also from healthy donor PBMCs (38) indicated that the *IL13RA1-LOR1a* isoform reached approximately 13% of canonical isoform expression (Fig. 1C), whereas the *IL13RA1-L2a* and *IL13RA1-AluJb* isoforms were much less abundant. To extend these findings, we analyzed short-read RNA-seq data, which may underestimate expression of RTE-exonizing *IL13RA1* isoforms. Nevertheless, canonical *IL13RA1* as well as all three alternative *IL13RA1* isoforms were detectable in RNA-seq from whole blood or isolated monocytes and B cells from healthy donors or patients with autoimmune or autoinflammatory conditions (39). This was not the case for CD4 T cells, which are known to lack *IL13RA1* expression (Fig. 1D). The canonical isoform was predominant and was expressed at higher levels in monocytes than in B cells, whereas the *IL13RA1-L2a* and *IL13RA1-AluJb* isoforms were the least expressed (Fig. 1D). *IL13RA1-LOR1a* expression varied the most. It approached the levels of the canonical isoform in certain donors, but was nearly undetectable in others (Fig. 1, D and E). Thus, considerable expression of alternative *IL13RA1* isoforms is detected in both full-length and short-read RNA-seq data under physiological conditions.

In comparison to the canonical IL-13Rα1 protein, all three RTE-exonizing *IL13RA1* isoforms encoded for C-terminally truncated products (fig. S2). The IL-13Rα1-L2a protein lacked most of the third fibronectin type-III domain and likely the ligand-binding ability of the canonical isoform. By contrast, IL-13Rα1-AluJb and IL-13Rα1-LOR1a retained the full extracellular domains. IL-13Rα1-AluJb was truncated at the start of the transmembrane helix, which is replaced by an alternative 15-amino acid sequence and is predicted to be a soluble form of the receptor. This resembles a soluble form of PD-L1, which is also generated by intronic RTE exonization (24). IL-13Rα1-LOR1a retained the transmembrane helix and proline-rich Box 1 domain in the cytoplasmic tail. However, it lacked the downstream phosphotyrosines and the Box 2 domain, which is implicated in TYK2 phosphorylation following cytokine binding (40, 41). These elements were instead replaced by an alternative 13-amino acid sequence (fig. S2). The splice junctions between canonical exons and RTE sequences in *IL13RA1-AluJb* and *IL13RA1-LOR1a* were confirmed by amplicon sequencing (fig. S3).

We focused on *IL13RA1-LOR1a*, given its higher expression among the three RTE-exonizing isoforms and its potential for signaling. Phylogenetic analyses suggested that the *LOR1a* and *L1MD1* elements co-opted in *IL13RA1-LOR1a* were acquired at the time of anthropoid divergence from prosimians, whereas the *AluJb* RTE was found in all primates (Fig. 1F). By contrast, the *L2a* element in *IL13RA1-L2a* appeared to be much older, tracing back to the origin of placental mammals (Fig. 1F). The *L1MD1* element diverged substantially from the consensus sequence, whereas the *LOR1a* element remained one of the best conserved within its group (87% and 79% identity with the *LOR1a* and *L1MD1* consensus sequences, respectively). Among anthropoids, motifs required for *LOR1a* and *L1MD1* exonization were highly conserved, whereas the coding *LOR1a* sequences were less conserved (Fig. 1, G and H). This suggested that removal of the cytoplasmic tail in anthropoid IL-13Rα1-LOR1a was the important selectable feature, whereas the specific sequence encoded by the *LOR1a* was less important. Consistent with conservation of the required motifs, the mRNAs of both *IL13RA1* and *IL13RA1-LOR1a* isoforms were readily detected by RT-qPCR in several nonhuman anthropoid cell lines, at a ratio comparable with that in human cells (Fig. 1I). Moreover, orthologous predicted *IL13RA1-LOR1a* transcripts were annotated in the chimpanzee (XM_016943711), bonobo (XM_034949439), and gorilla (XM_019019634). Thus, alternative *IL13RA1* isoforms created by RTE exonization have the potential to affect signaling by type 2 cytokines in human cells and those of other primates.

### *IL13RA1* transcription linked with male B cell activation

**Fig. 2:**
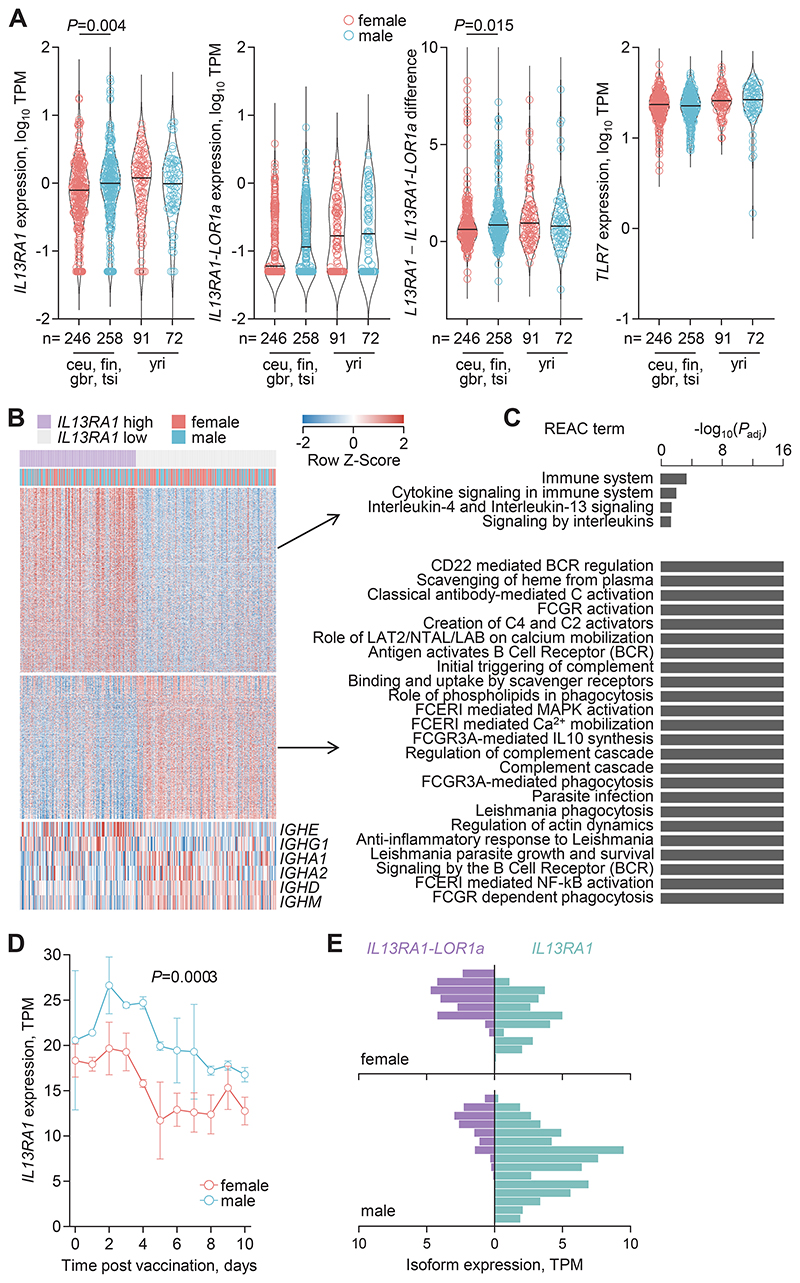
*IL13RA1* transcription linked with male B cell activation. (**A**) Expression of *IL13RA1* and *IL13RA1-LOR1a*, their difference, and expression of *TLR7* in RNA-seq data from LCLs from healthy individuals (PRJEB3366), according to donor sex and ancestry. Number of independent donors are indicated in the figure and *P* values were calculated with Mann–Whitney *U* tests between male (blue) and female (red) donors. (**B**) Heatmap of differential gene expression (≥ 2-fold change; *P* ≤ 0.05; q ≤ 0.05) between LCLs (PRJEB3366) with high (≥ 2 TPM, *n* = 131) and low (≤ 0.4 TPM, *n* = 158) *IL13RA1* expression. Differentially expressed immunoglobulin heavy chain genes are also depicted separately. The enrichment of male and female donors in *IL13RA1*^hi^ and *IL13RA1*^lo^ subsets, respectively, is significant (*P* = 0.018, Fisher’s exact test). (**C**) Functional annotation by reactome terms (REAC) of the genes overlapping the most up-regulated and down-regulated transcripts (*n* = 100 each), respectively, from (B). *P* values were calculated with the g:SCS algorithm. (**D**) *IL13RA1* expression in RNA-seq data from PBMCs isolated from healthy male (*n* = 2) or female donors (*n* = 3) on consecutive days following influenza vaccination (E-GEOD-45735). The *P* value was calculated with a two-tailed Student’s *t* test of the area under the curve. (**E**) Mirror plots of *IL13RA1* and *IL13RA1-LOR1a* expression in RNA-seq data for peripheral blood B cells isolated from allergic and non-allergic male (*n* = 15) and female adolescents (*n* = 12) (GSE165316). *IL13RA1* expression and the difference in isoform expression are significantly higher in male than in female B cells (*P* = 0.022 and *P* = 0.0031, respectively; Student’s *t* test). Each bar represents an individual donor.

As IL-13Rα1-LOR1a may be functionally distinct from the canonical isoform, we next quantified their expression separately in RNA-seq data from LCLs. Similar to our analysis of microarray data (fig. S1, B and C), this analysis showed significantly higher *IL13RA1* expression in male compared to female LCLs of European ancestry, but not in those of African ancestry (Fig. 2A). The expression of both *IL13RA1-LOR1a* (and *TLR7*) was comparable between male and female LCLs and was independent of ancestry, (Fig. 2A). The difference in *IL13RA1* and *IL13RA1-LOR1a* expression was therefore significantly higher in male than in female LCLs of European ancestry (Fig. 2A).

To explore possible mechanisms underlying the observed patterns in *IL13RA1* expression, we compared the transcriptional profiles of LCLs with high (≥ 2 transcripts per million (TPM)) and low (≤ 0.4 TPM) *IL13RA1* expression. This analysis identified 1888 transcripts with significantly different expression (Fig. 2B and data file S1). *IL13RA1*^hi^ and *IL13RA1*^lo^ subsets were also enriched in male and female donors, respectively (Fig. 2B), further underscoring that males express higher *IL13RA1* levels. Differentially expressed transcripts were not enriched in X-linked genes (data file S1), but instead associated with distinct biological pathways. Transcripts overexpressed in the *IL13RA1*^hi^ LCLs were associated primarily with IL-4 and IL-13 signaling, whereas those overexpressed in the *IL13RA1*^lo^ LCLs showed much stronger associations with pathways related to activation and signaling by the B cell receptor (BCR), Fc and other immunoglobulin (Ig) receptors, and complement (Fig. 2C). Further supporting differential involvement of the BCR, *IL13RA1*^hi^ LCLs transcribed higher levels of *IGHE* and *IGHG1*, a class that is expressed in the precursors of high affinity IgE plasma cells (42), whereas *IL13RA1*^lo^ LCLs transcribed higher levels of unswitched *IGHD* and *IGHM* and class-switched *IGHA1* and *IGHA2*, but not other switched classes (Fig. 2B). These results linked higher *IL13RA1* expression with preferential switch to IgG1 and IgE and with a distinct state of activation in response to cytokines rather than the BCR. By extension, male LCLs, which were more likely to express higher *IL13RA1* levels, were also more likely to transcribe *IGHG1* and *IGHE* and adopt this distinct state of activation. These results are also consistent with the stepwise increase in IL-13Rα1 expression that has been observed in IgG1 and IgE class-switched germinal center (GC) B cells, compared with unswitched IgM GC B cells, following mouse immunization with a model type 2 allergen (43).

To extend this potential link to primary cells, we measured *IL13RA1* expression in RNA-seq data from male and female healthy donor PBMCs over the course of influenza A virus (IAV) vaccination (44). Although genetic variants in type 2 cytokines and receptors may affect vaccine responsiveness (45, 46), IAV vaccination was chosen here as an in vivo model for primary B cell activation. Despite the small sample size, male PBMCs showed significantly higher *IL13RA1* expression in response to vaccination and throughout the observation period than female PBMCs (Fig. 2D). Moreover, *IL13RA1* expression in RNA-seq data from B cells isolated from adolescents with or without IgE-mediated food allergies (47) was significantly higher in male than in female B cells (Fig. 2E). By contrast, expression of *IL13RA1-LOR1a* tended to be higher (although not statistically significantly) in female B cells, and the difference in isoform expression was significantly lower in female than in male B cells (Fig. 2E). Thus, B cells from males express higher levels of *IL13RA1* than those from females, which correlates with a state of heightened activation and IgG1 and IgE class switching. Although differences between the sexes may be amplified by B cell differentiation to IgE^+^ clones in males, our model suggests that differential baseline expression of the two *IL13RA1* isoforms is a contributor to subsequent responses.

As the observed sex bias in *IL13RA1* expression contrasted with the expected effect of escape from X inactivation, we explored whether it was controlled by sex hormones. LCLs exhibited substantial expression of estrogen receptor 1 (ESR1), but not of androgen receptor (AR) (fig. S4A). Moreover, *IL13RA1* expression correlated positively with expression of the ESR1 target gene *IGFBP4* (fig. S4A). *IGFBP4* also displayed sex bias in expression (fig. S4B). To exclude the effect of biological sex, male and female LCLs were considered separately. This showed significantly higher expression of *ESR1* specifically in male LCLs with lower *IL13RA1* expression (fig. S4C). Similar results were obtained from an analysis of data from Cancer Cell Line Encyclopedia (CCLE) hematopoietic cell lines (48) (fig. S4, D and E). Both ESR1 and AR were found to bind the *IL13RA1* promoter (fig. S5A). Moreover, treatment of Hodgkin lymphoma L-1236 cell line (selected for its relatively high *IL13RA1* expression) with the estrogen steroid hormone 17β-estradiol significantly reduced *IL13RA1* mRNA levels and total IL-13Rα1 protein levels, without affecting *IL13RA1-LOR1a* mRNA levels (fig. S5, B and C), further supporting ESR1 as an *IL13RA1* repressor. Analysis of RNA-seq data from PBMCs isolated from healthy male and female donors across the age spectrum (49) revealed significantly lower expression specifically of the canonical *IL13RA1* isoform in premenopausal females (< 45 years of age), compared with age-matched males, but not in older-age females (> 50 years of age) (fig. S5D), consistent with a possible effect of estrogen. Thus, biological sex (and sex hormones in particular) primarily affect the expression of the canonical *IL13RA1* isoform. However, expression of the alternative *IL13RA1-LOR1a* isoform exhibits greater variation among healthy individuals seemingly independently of biological sex.

We therefore explored additional factors potentially influencing the balance of these two isoforms. The full-length *IL13RA1* 3′ untranslated region (UTR) contains multiple AU-rich element (ARE) motifs (fig. S6A), previously implicated in the regulation of *IL13RA1* mRNA stability (50, 51). It also contains three alternative polyadenylation signals, creating *IL13RA1* isoforms with different 3′ UTR lengths, which were supported by full-length RNA-seq data from healthy donor PBMCs (fig. S6, A to C). The alternative splicing that generates the *IL13RA1-LOR1a* isoform removes these AREs, releasing it from regulation by ARE-binding proteins. Accordingly, overexpression of the ARE-binding protein tristetraprolin (TTP) (encoded by *ZFP36*) in the LK-2 cell line partially reduced the abundance of canonical *IL13RA1*, but not *IL13RA1-LOR1a* mRNAs (fig. S6D). Moreover, expression of *ZFP36*, as well as of annotated TTP target genes (51), negatively correlated with *IL13RA1* isoform expression in LCLs, independent of biological sex (figs. S7). By contrast, expression of other *IL13RA1* mRNA stability regulators, including microRNAs *MIR31* and *MIR155* (52) and the circular RNA *circPAN3* (53), showed no correlation (figs. S8).

*IL13RA1* expression can also be induced or repressed by IL-13Rα1 signaling itself in a cell type– specific manner (54–56). In turn, genetic variation in cytokine, receptor or adaptor genes, linked with IgE sensitization and development of allergic disease, as well as with genetic ancestry (5, 57, 58), can affect the strength of type 2 cytokine signaling. By calling single-nucleotide polymorphisms (SNPs) (59) from RNA-seq data, we accordingly found that genetic variation in *STAT6*, but not other type 2 immune genes, was associated with a lower *IL13RA1-LOR1a*:*IL13RA1* ratio in male donor PBMCs (fig. S9). Thus, each *IL13RA1* isoform is regulated by multiple independent mechanisms, whose integration would determine the isoform ratio.

### Reduced signaling by the IL-13Rα1-LOR1a isoform

**Fig. 3:**
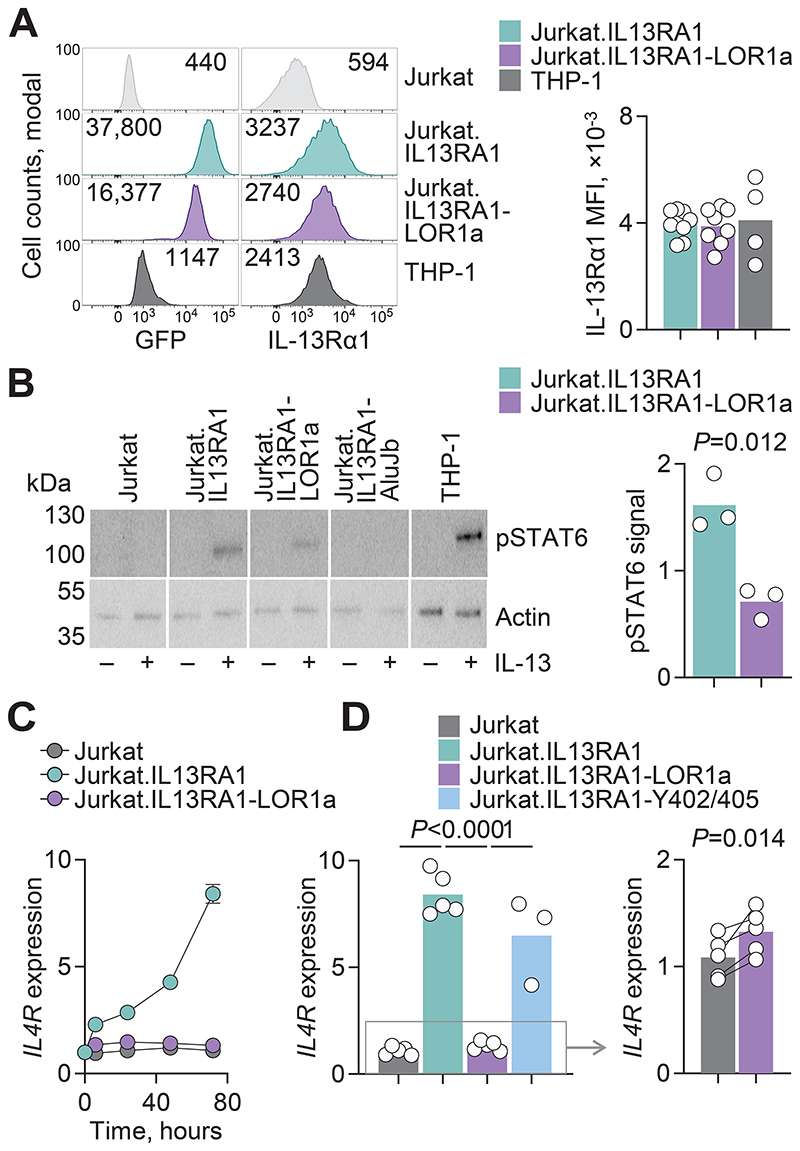
Partially defective IL-13Rα1-LOR1a signaling in a T cell line. (**A**) Left: representative flow cytometry plots (left) of IL-13Rα1 and GFP reporter expression in parental Jurkat cells or Jurkat cells transduced to express IL-13Rα1 (Jurkat.IL13RA1) or IL-13Rα1-LOR1a (Jurkat.IL13RA1-LOR1a) and GFP. Right: quantitation of these flow cytometry data. Endogenous IL-13Rα1 expression in the monocytic cell line THP-1 is also included as a control. Numbers denote the geometric mean fluorescence intensity (MFI) and symbols represent the pooled independent experiments (*N* = 9 for Jurkat, *N* = 4 for THP-1). (**B**) Left: protein immunoblot for pSTAT6 and loading control (Actin) in the same cell lines as in (A), before and after stimulation with IL-13. Jurkat cells transduced to express IL-13Rα1-AluJb (Jurkat.IL13RA1-AluJb) are also included as control. Right: quantitation of pSTAT6 signal relative to Actin signal following IL-13 stimulation of Jurkat.IL13RA1 and Jurkat.IL13RA1-LOR1a. Symbols represent the pooled independent experiments (*N* = 3) and the *P* value was calculated with a paired two-tailed Student’s *t* test. (**C**) Mean ± SEM (*N* = 1 to 5) expression of *IL4R*, determined by RT-qPCR and plotted relative to *HPRT1* expression, over the course of IL-13 stimulation of the same cell lines as in (A). (**D**) Left: *IL4R* expression, determined as in (C), 72 hours after IL-13 stimulation of the indicated cell lines. Jurkat cells transduced to express IL-13Rα1 with mutations in the intracellular tail tyrosines (Jurkat. IL13RA1-Y402/405F) are also included. Symbols represent the pooled independent experiments (*N* = 3 to 5) and *P* values were calculated with one-way ANOVA. Right: the same data as in the left, plotted on a different scale to visualize the difference between Jurkat and Jurkat.IL13RA1-LOR1a cells. The *P* value was calculated with a paired two-tailed Student’s *t* test.

To understand the functional implications of IL-13Rα1-LOR1a isoform expression, we next examined its signaling capacity. We first used the Jurkat T cell line, which lacks *IL13RA1*, but expresses all other signaling components (fig. S10). We established Jurkat clones engineered to express either IL-13Rα1 or IL-13Rα1-LOR1a at levels comparable between them and also comparable With endogenous IL-13Rα1 expression in the monocyte cell line THP-1 (Fig. 3A). Stimulation of both these clones and of THP-1 cells with IL-13, but not of parental Jurkat cells or Jurkat cells expressing the IL-13Rα1-AluJb isoform, induced STAT6 phosphorylation (Fig. 3B), demonstrating that both IL-13Rα1 and IL-13Rα1-LOR1a can form an IL-13–binding functional receptor with signaling capacity. However, STAT6 phosphorylation was significantly reduced in Jurkat.IL13RA1-LOR1a cells to an average of 44% of that in Jurkat.IL13RA1 cells (Fig. 3B). *IL4R* itself is a STAT6 target gene in T cells and its transcription was elevated eightfold by IL-13 treatment in Jurkat.IL13RA1 cells (Fig. 3C). Conversely, a statistically significant 1.2-fold *IL4R* induction was detected in Jurkat.IL13RA1-LOR1a cells after IL-13 treatment (Fig. 3D). Thus, the IL-13Rα1-LOR1a isoform exhibited only a partial defect in proximal STAT6 phosphorylation, but a more severe defect in downstream target gene transcription. Prior studies with artificial truncations of the IL-13Rα1 subunit coincidentally similar to the natural IL-13Rα1-LOR1a isoform demonstrated a defect specifically in TYK2 phosphorylation and subsequent activation (40, 41). Mutation of the two phosphotyrosines in the IL-13Rα1 cytoplasmic tail did not compromise responsiveness of Jurkat.IL13RA1-Y402/405F cells to IL-13 (Fig. 3D), whereas mutation of phosphotyrosines in the IL-4Rα cytoplasmic tail abolished the responsiveness of HeLa cells (fig. S11). These findings support a model of TYK2 activation by the IL-13Rα1 subunit and tyrosine phosphorylation in the IL-4Rα subunit for maximal responsiveness to IL-13.

**Fig. 4:**
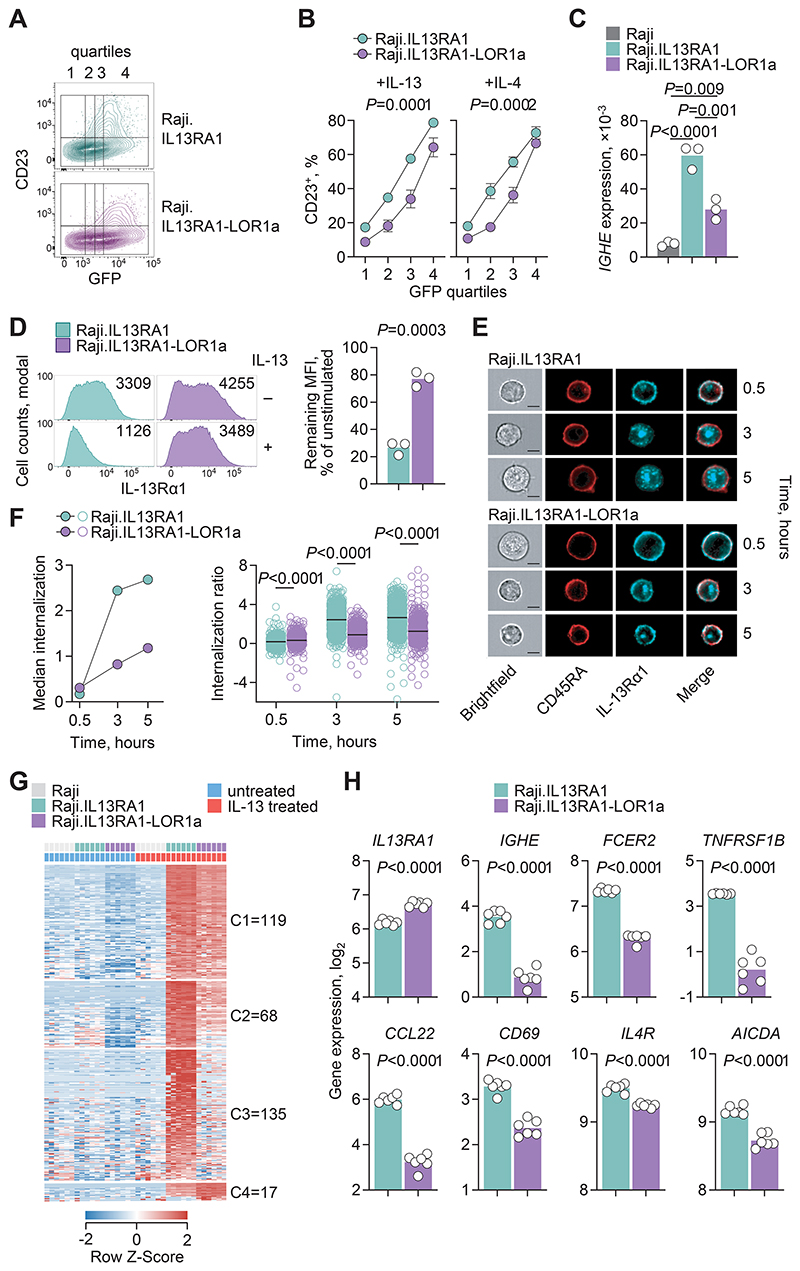
Partially defective IL-13Rα1-LOR1a signaling in a B cell line. (**A**) Representative flow cytometry plots of GFP expression quartiles and CD23 expression following IL-13 treatment of Raji cells transduced to express IL-13Rα1 (Raji.IL13RA1) or IL-13Rα1-LOR1a (Raji.IL13RA1-LOR1a) and GFP. (**B**) Mean ± SEM percentage from 3 independent experiments (*N* = 3) of CD23^+^ cells in each GFP expression quartile in the same cells as in (A) following treatment with IL-13 or IL-4. (**C**) *IGHE* expression, determined by RT-qPCR and plotted relative to *HPRT1* expression, in the same cells as in (A), following treatment with IL-13. Symbols represent the pooled independent experiments (*N* = 3) (**D**) Left: Representative flow cytometric detection of IL-13Rα1 expression in the same cells as in (A) with and without IL-13 treatment. Numbers denote the MFI. Right: MFI quantitation of IL-13Rα1 expression in IL-13–stimulated relative to unstimulated cells. (C and D) Symbols represent the pooled independent experiments (*N* = 3) and *P* values were calculated with (C) one-way ANOVA or (D) two-tailed Student’s *t* tests. (**E**) Representative images of CD45RA and IL-13Rα1 localization over time following IL-13 treatment of Raji.IL13RA1 and Raji.IL13RA1-LOR1a cells (scale bar: 7 μm). (**F**) Left: median IL-13Rα1 internalization in the indicated cell population. Right: IL-13Rα1 internalization ratio in individual cells (*n* = 1000) from the same cell populations in (E). (**G**) Heatmap of expression of genes significantly induced (≥ 2-fold change; *P* ≤ 0.05; q ≤ 0.05) upon IL-13 treatment of Raji.IL13RA1 or Raji.IL13RA1-LOR1a cells. Columns represent independent replicates of each condition. (**H**) Expression of the indicated gene in the same samples as in (F). Symbols are combined values (*n* = 6) of *n* = 2 technical replicates per group per experimental replicate, with *N* = 3 independent experimental replicates. *P* values were calculated with two-tailed Student’s *t* tests.

To examine the capacity of IL-13Rα1-LOR1a to mediate type 2 B cell responses, such as transcription of the IgE prerequisite *IGHE* and expression of the low-affinity IgE receptor CD23, we used Raji B cells, which also lack endogenous *IL13RA1* expression. Raji cells transduced to express IL-13Rα1 initiated *IGHE* transcription and expressed CD23 in response to IL-4 or IL-13, directly proportional to the levels of IL-13Rα1 (fig. S12, A to C), consistent with a rate-limiting function of IL-13Rα1 in this setting. We then established a clonal Raji population expressing the IL-13Rα1-LOR1a isoform at similar or higher levels than a clonal Raji population expressing the canonical IL-13Rα1 isoform (fig. S12D). Despite its higher expression, IL-13Rα1-LOR1a was unable to mediate CD23 expression or *IGHE* transcription in response to IL-4 or IL-13 as effectively as IL-13Rα1 (Fig. 4, A to C). Moreover, the degree and rate of receptor complex internalization following cytokine treatment, a prerequisite of signal amplification (60), was significantly lower for IL-13Rα1-LOR1a than for the canonical isoform (Fig. 4, D to F). Thus, the reduced capacity for internalization may be a contributor to defective signaling initiated by the IL-13Rα1-LOR1a isoform.

For a more comprehensive view of IL-13Rα1-LOR1a signaling, we compared the transcriptional response of Raji.IL13RA1 and Raji.IL13RA1-LOR1a cells to IL-13. Approximately one third of the genes that were significantly induced by IL-13 in Raji.IL13RA1 cells were comparably induced also in Raji.IL13RA1-LOR1a cells (Fig. 4G), highlighting residual signaling capacity of IL-13Rα1-LOR1a. By contrast, the majority of genes induced by IL-13 in Raji.IL13RA1 cells were significantly less inducible or completely unresponsive in Raji.IL13RA1-LOR1a cells (Fig. 4G). Genes involved in type 2 B cell responses, including *IGHE, FCER2, IL4R*, and *AICDA*, were induced at significantly higher levels by IL-13Rα1 than IL-13Rα1-LOR1a (Fig. 4H). In this experimental system, transcription of *IL13RA1* was significantly higher in Raji.IL13RA1-LOR1a than in Raji.IL13RA1 cells, ruling out the possibility that the defective up-regulation of target genes was caused by insufficient expression of IL-13Rα1-LOR1a. Thus, the IL-13Rα1-LOR1a isoform exhibits partial signaling defects, consistent with defects described for artificial truncations of the IL-13Rα1 intracellular tail (40, 41).

### Antagonistic function of the IL-13Rα1-LOR1a isoform

Integration of the *LOR1a* and *L1MD1* elements and their subsequent exonization diverts splicing of the *IL13RA1* transcript from its canonical terminal exon. This reduces canonical *IL13RA1* splicing and, by extension, overall responsiveness to IL-4 and IL-13 as a transcriptional effect of retroviral integration. However, the signaling defects of IL-13Rα1-LOR1a also raise the possibility that it may act as an antagonist of the canonical signaling–competent isoform at the protein level, by competing for IL-4, IL-13, or any signaling components of the receptor complex. Considering our observation that responses to IL-4 and IL-13 may be directly limited by the availability of IL-13Rα1, potential antagonism by IL-13Rα1-LOR1a may have a dominant-negative effect, which would depend on the relative ratio of the two isoforms.

**Fig. 5:**
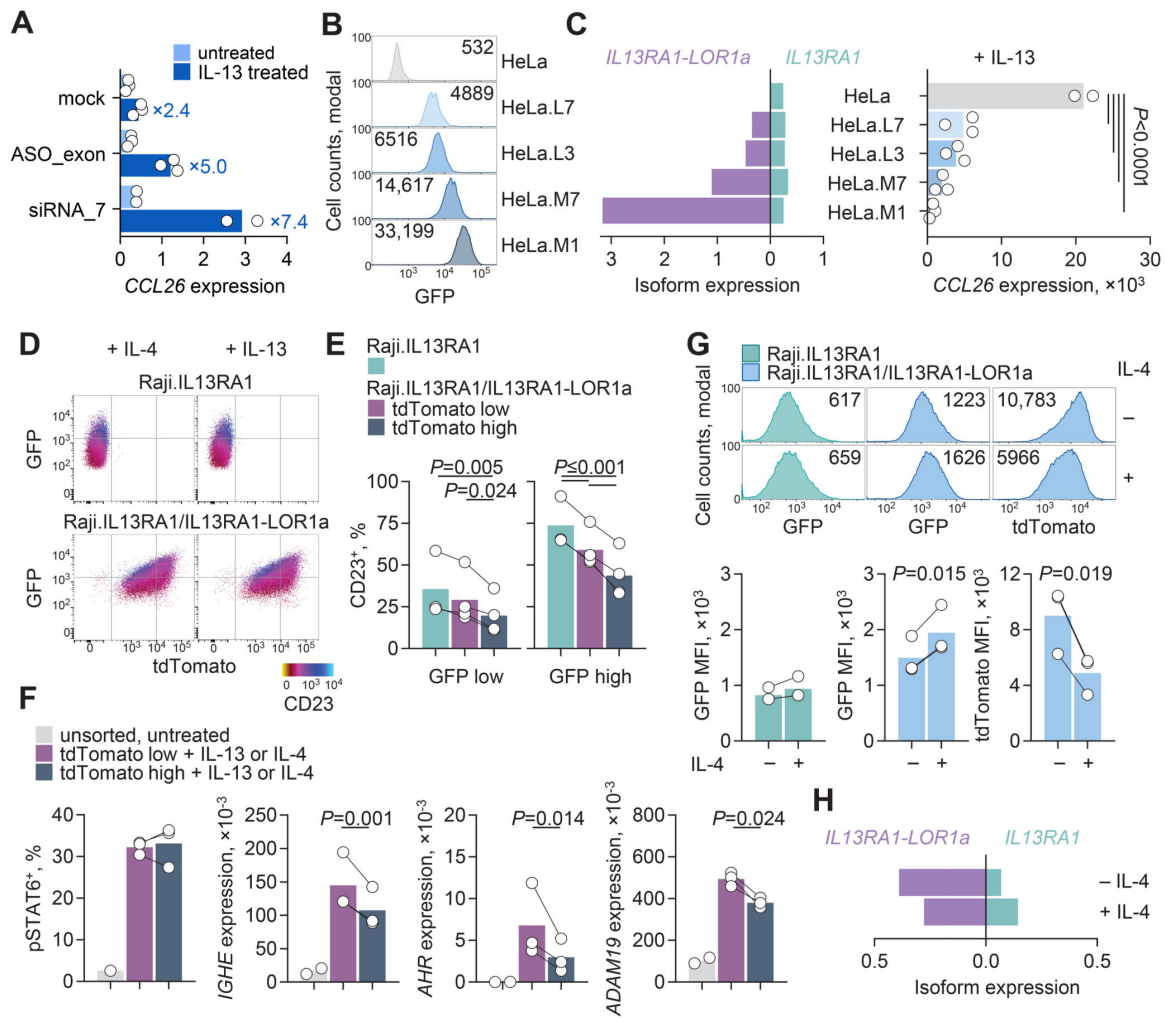
IL-13Rα1-LOR1a antagonizes IL-13Rα1 signaling. (**A**) *CCL26* expression by RT-qPCR relative to *HPRT1* expression in U2OS cells mock-transfected or transfected with *LOR1a* exon-targeting ASOs or siRNA, then treated with IL-13 or untreated. Symbols represent the pooled independent experiments (*N* = 2 to 3) and numbers denote *CCL26* fold induction in IL-13–treated versus untreated cells. (**B**) Representative flow cytometry of GFP expression in parental and HeLa cell clones transduced to express *IL13RA1-LOR1a* and GFP. Numbers denote GFP MFI. (**C**) Left: *IL13RA1* and *IL13RA1-LOR1a* expression by RT-qPCR relative to *HPRT1* expression, in the same cells as in (B) (*N* = 1). Right: *CCL26* expression by RT-qPCR relative to *HPRT1* expression, in the same cells following treatment with IL-13. Symbols represent the pooled independent experiments (*N* = 2 to 3) and *P* values calculated with one-way ANOVA. (**D**) Representative expression of CD23 by flow cytometry, indicated by a heatmap, following IL-4 or IL-13 treatment of Raji cells transduced to express IL-13Rα1 reported by GFP (Raji.IL13RA1), or with IL-13Rα1-LOR1a reported by tdTomato (Raji.IL13RA1/IL13RA1-LOR1a). (**E**) Percentage CD23^+^ cells following IL-13 treatment of the same cells as in (D), according to GFP and tdTomato expression. (**F**) Percentage of pSTAT6^+^ cells by flow cytometry, and *IGHE, AHR* and *ADAM19* expression by RT-qPCR relative to *HPRT1* expression, following IL-13 (pSTAT6) or IL-4 (gene expression) of the same cells as in (D). (**G**) Top: representative flow cytometry plots of GFP and tdTomato expression in the same cells as in (D), with or without IL-4 treatment. Bottom: GFP and tdTomato MFI in the same cells. (E to G) Symbols represent the pooled independent experiments (*N* = 1 to 4) and *P* values were calculated with (E) one-way repeated measures ANOVA with Bonferroni correction for multiple comparisons or (F and G) two-tailed Student’s paired *t* tests. (**H**) *IL13RA1* and *IL13RA1-LOR1a* expression by RT-qPCR relative to *HPRT1* expression, in the same cells as in (D), with or without IL-4 treatment (*N* = 1).

To examine whether endogenous expression of *IL13RA1-LOR1a* can affect the ability of the cells to sense IL-4 or IL-13, we targeted its expression in U2OS osteosarcoma cells, which respond to stimulation by up-regulating *CCL26*. We selected antisense oligonucleotides (ASOs) or small interfering RNA (siRNA) oligonucleotides that efficiently reduced the expression of *IL13RA1-LOR1a* without affecting the canonical (fig. S13A). The selected ASO and siRNA oligonucleotides augmented the responsiveness of U2OS cells to IL-13, by twofold and threefold, respectively (Fig. 5A), in line with their knockdown efficiency (fig. S13A).

For orthogonal validation, we used CRISPR/Cas9–mediated deletion of the *LOR1a* element in NCI-H358 lung adenocarcinoma cells. IL-13 treatment of these cells—which express a typical *IL13RA1* isoform ratio—caused significant down-regulation specifically of the canonical *IL13RA1* isoform (fig. S13B). CRISPR/Cas9–mediated deletion of the *LOR1a* element (fig. S13C) also affected the levels of the canonical *IL13RA1* isoform, both at the bulk and clonal population levels, indicating a role for *IL13RA1-LOR1a* in fine-tuning IL-13Rα1 responsiveness. Despite the reduction in *IL13RA1* expression, which would have reduced responsiveness to IL-13, the *IL13RA1-LOR1a*-deficient NCI-H358 clone expressed higher levels of *CCL26* than the control clone in response to IL-13 (fig. S13E). Thus, the *IL13RA1-LOR1a* isoform appears to play a role in the fine-tuning of IL-13Rα1 responsiveness.

We next examined the effects of increased *IL13RA1-LOR1a* expression. To this end, we used HeLa cells, as they express almost exclusively the canonical *IL13RA1* isoform. Parental HeLa cells were compared with clones transduced to additionally express *IL13RA1-LOR1a* at different ratios relative to the canonical (ranging from 1:1 to 1:13) (Fig. 5, B and C). Expression of *IL13RA1-LOR1a* in HeLa cells did not appreciably affect transcription of endogenous *IL13RA1* and led to a substantial reduction in *CCL26* induction by IL-13 already at the lowest level (1:1 ratio) and further reduction upon increasing *IL13RA1-LOR1a* levels (Fig. 5C). IL-13 failed to induce *CCL26* in HeLa cells overexpressing *IL13RA1-LOR1a* at levels 13 times higher than *IL13RA1* (Fig. 5C). Thus, the inhibitory effect of IL-13Rα1-LOR1a on IL-13Rα1-mediated signaling dominates over any residual signaling activity of IL-13Rα1-LOR1a itself.

To test the IL-13Rα1-LOR1a potential for antagonism in B cells, we coexpressed the *IL13RA1* and *IL13RA1-LOR1a* isoforms in Raji cells, with their relative levels reported by bicistronically expressed GFP and tdTomato, respectively. Treatment with IL-4 or IL-13 elicited the strongest CD23 up-regulation in cells with the lowest *IL13RA1-LOR1a:IL13RA1* ratio, whereas the weakest responses were produced by cells with the highest *IL13RA1-LOR1a:IL13RA1* ratio (Fig. 5, D and E). Indeed, compared with Raji.IL13RA1 control cells expressing equivalent levels of GFP, CD23 induction was significantly curtailed in Raji.IL13RA1/IL13RA1-LOR1a cells with low tdTomato expression and even more curtailed in those with high tdTomato expression (Fig. 5E), revealing a dose-dependent inhibitory effect of the IL-13Rα1-LOR1a isoform. Similar results were obtained with Raji cells transduced with vectors encoding *IL13RA1* or *IL13RA1-LOR1a* together with the same reporter gene (fig. S14). In Raji.IL13RA1/IL13RA1-LOR1a cells first purified according to tdTomato expression (fig. S15) and then treated with IL-13 or IL-4, early STAT6 phosphorylation was similar between the two subsets with high and low tdTomato expression (Fig. 5F). Nevertheless, transcription of exemplar genes, such as *IGHE, AHR* and *ADAM19* was significantly lower at later time points (96 hours) in cells with high than with low tdTomato expression (Fig. 5F), in agreement with CD23 protein expression. Thus, IL-13Rα1-LOR1a antagonism causes substantial defects in downstream IL-13Rα1 signaling, which may not be entirely predicted by proximal STAT6 phosphorylation.

Type 2 cytokine signaling can induce CD23 expression and *IGHE* transcription. It can also promote the survival and proliferation of responsive B cells (33). We therefore examined the effect of the IL-13Rα1-LOR1a isoform on these parameters in Raji cells treated with IL-4. In Raji populations expressing only the canonical *IL13RA1* isoform, IL-4 treatment selected for cells with higher GFP levels (reporting *IL13RA1* expression) (Fig. 5G). A similar proliferative advantage of cells expressing higher levels of IL-13Rα1 (reported by GFP) and lower levels of IL-13Rα1-LOR1a (reported by tdTomato) was seen also in Raji cells coexpressing both isoforms (Fig. 5G), where IL-4 treatment additionally shifted the ratio of the two isoforms at the mRNA level in favor of canonical *IL13RA1* transcription (Fig. 5H). Thus, IL-13Rα1-LOR1a may also attenuate the proliferative effect of type 2 cytokine signaling, with counterselection of cells with an unfavorable *IL13RA1-LOR1a*:*IL13RA1* ratio.

**Fig. 6:**
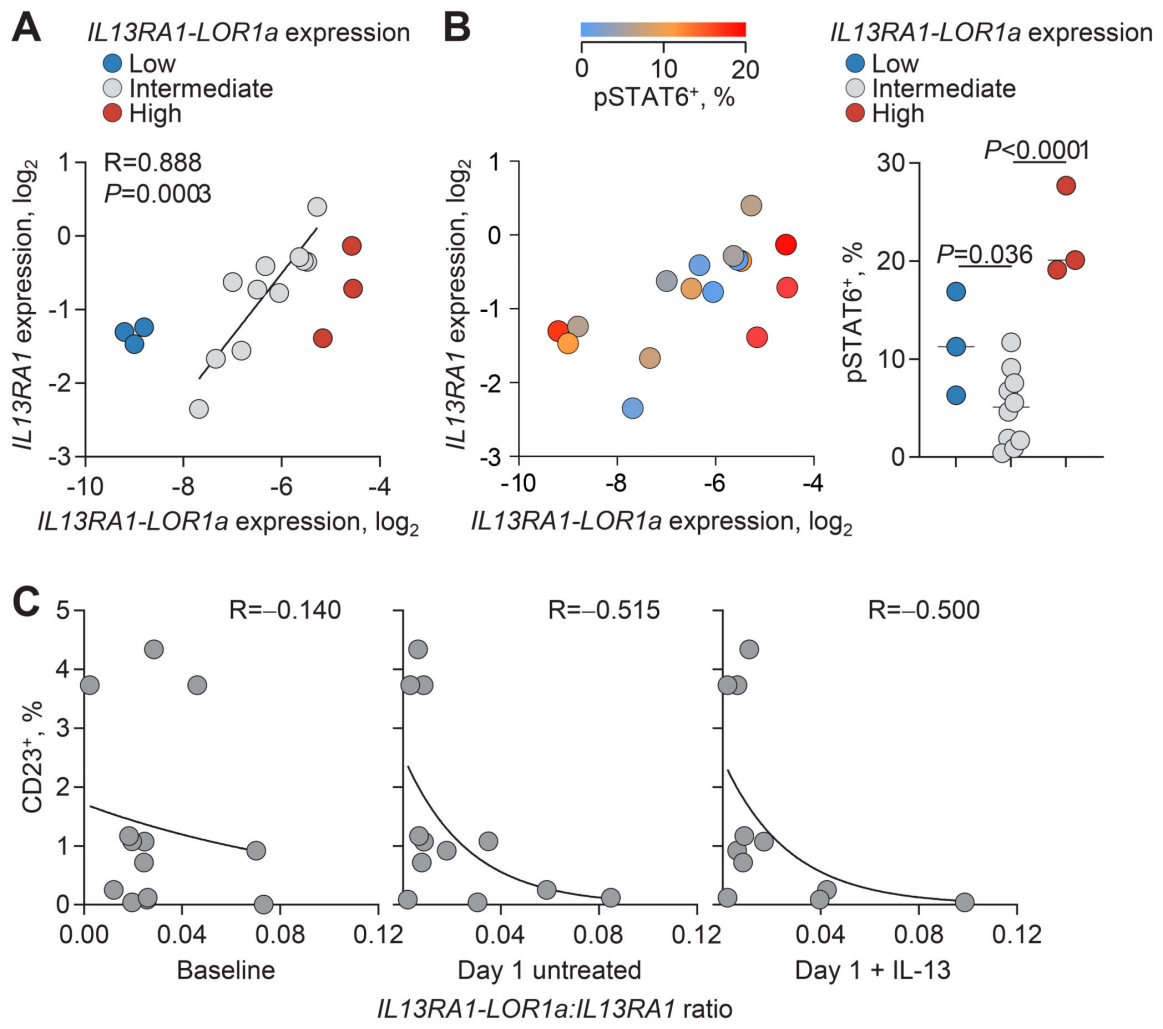
IL-13Rα1-LOR1a antagonism of IL-13Rα1 signaling in primary human B cells. (**A**) *IL13RA1* and *IL13RA1-LOR1a* expression, determined by RT-qPCR relative to *HPRT1* expression, in primary human B cells. (**B**) Left: percentage of pSTAT6^+^ cells (indicated by a heatmap) 30 min after IL-13 treatment of purified primary human B cells, according to baseline *IL13RA1* and *IL13RA1-LOR1a* expression as in (A). Right: percentage of pSTAT6^+^ cells in same IL-13-treated primary human B cells from donors stratified according to baseline *IL13RA1-LOR1a* expression. *P* values were calculated with a one-way ANOVA using the intermediate group as the control and Šídák correction for multiple comparisons. (**C**) Percentage of CD23^+^ cells 1 day after IL-13 treatment of purified primary human B cells, according to *IL13RA1* and *IL13RA1-LOR1a* expression at baseline and following 1 day of culture with or without addition of IL-13. (A to C) Each symbol represents purified primary B cells from an individual healthy human donor (*n* = 17).

We next investigated whether IL-13Rα1-LOR1a also tunes type 2 cytokine signaling in primary B cells. To this end, we isolated peripheral blood B cells from healthy donors and compared the balance of *IL13RA1* and *IL13RA1-LOR1a* expression directly ex vivo. In agreement with RNA-seq data analysis (Fig. 1E), *IL13RA1-LOR1a* mRNA levels varied considerably. In most healthy donors, expression of the two isoforms was correlated (Fig. 6A). However, in certain individuals, *IL13RA1-LOR1a* transcription was found at either very high or very low levels, irrespective of *IL13RA1* transcription (Fig. 6A). Brief treatment of purified B cells with IL-13 induced variable levels of STAT6 phosphorylation (fig. S16, A and B). The frequency of phosho-STAT6 (pSTAT6)^+^ cells was the highest in B cells at both extremes of *IL13RA1-LOR1a* expression (Fig. 6B). High levels of pSTAT6 following IL-13 treatment might have been expected in cells with the highest combined *IL13RA1* isoform expression given that the *IL13RA1-LOR1a* isoform could initiate STAT6 phosphorylation in Jurkat (Fig. 3B) and Raji cells (Fig. 5F). However, primary B cells with low *IL13RA1-LOR1a* expression exhibited higher levels of pSTAT6 than would be expected based on their *IL13RA1* expression and significantly more than cells with intermediate expression of both isoforms (Fig 6B), suggesting an inhibitory effect of the IL-13Rα1-LOR1a isoform.

As a measure of downstream responses of primary B cells to type 2 cytokines, we also examined induction of CD23 expression and *IGHE* transcription at later time points. IL-13 treatment alone was insufficient to induce *IGHE* transcription under these conditions, in agreement with previous work (33). Also consistent with prior reports (61), CD23 was expressed at highly variable levels in freshly isolated B cells, but was quickly down-regulated upon in vitro culture in the absence of stimulation (fig. S16C). IL-13 treatment induced CD23 expression in a proportion of B cells (fig. S16C) and the difference in CD23 expression between IL-13–treated and –untreated cultures was therefore considered a readout of IL-13 responsiveness. IL-13 treatment–induced CD23 expression levels exhibited a negative correlation with the *IL13RA1-LOR1a*:*IL13RA1* ratio (Fig. 6C), again suggesting an inhibitory effect of the IL-13Rα1-LOR1a isoform. Moreover, B cells from two of the three donors with the highest *IL13RA1-LOR1a* expression and pSTAT6 levels, failed to up-regulate CD23 expression (Fig. 6C). Thus, early STAT6 phosphorylation does not accurately predict subsequent responses, in agreement with similar findings in Jurkat and Raji cell lines.

Expression of *IL13RA1* and other signaling components may also be modulated in response to type 2 cytokines (54), thus modifying further responsiveness to such cytokines. Indeed, expression of both *IL13RA1* and *IL13RA1-LOR1a* was strongly up-regulated in primary B cells from the majority of donors over the course of IL-13 treatment. A smaller but still significant up-regulation of *IL13RA1* and *IL13RA1-LOR1a* was also observed in the absence of treatment (fig. S17A). Modulation of isoform expression also affected their ratio. The *IL13RA1-LOR1a*:*IL13RA1* ratio increased over time in the majority of samples, particularly those with balanced baseline isoform expression. By contrast, this ratio was rapidly reduced in those with a high baseline ratio and remained low in those with a low baseline ratio (fig. S17B). IL-13 treatment–induced CD23 expression levels accordingly exhibited a stronger negative correlation with the concurrent than the baseline *IL13RA1-LOR1a*:*IL13RA1* ratio (Fig. 6C). Thus, the dynamic modulation of isoform expression during stimulation appears to further tune responsiveness to continuous IL-13 stimulation.

## Discussion

Cytokine activity underpins cellular communication during the immune response and requires effective regulation to mediate host protection while minimizing autoimmunity or allergy. We identified an isoform of the common IL-4 and IL-13 type II receptor IL-13Rα1 subunit in anthropoid primates, which is partially defective in transmitting type 2 cytokine signals and can antagonize the canonical receptor. This isoform is referred to here as IL-13Rα1-LOR1a, because it is generated via the use of a *LOR1a* and an adjacent *L1MD1* retroelement downstream of the *IL13RA1* locus, which is employed as an alternative terminal exon. Such replacement of the canonical *IL13RA1* terminal exon has notable consequences both for the alternative mRNA and protein isoform.

The translated IL-13Rα1-LOR1a protein lacks important motifs in the cytoplasmic tail, which are essential for its full signaling capacity. The requirement for heterodimerization with the IL-4Rα subunit allows for the possibility of competition between the IL-13Rα1 isoforms. Combined with its reduced signaling capacity, the IL-13Rα1-LOR1a isoform may, in principle, antagonize the function of the canonical isoform. Indeed, selective knockdown of *IL13RA1-LOR1a* or coexpression of the two isoforms at physiological ratios—as we have employed here in U2OS, Raji, or HeLa cells—does support such antagonism. These data are further bolstered by parallel findings in primary B cells. The balance of the *IL13RA1* and *IL13RA1-LOR1a* isoforms is rather fixed in engineered cell lines, but is highly dynamic in primary cells. The balance between *IL13RA1* and *IL13RA1-LOR1a* mRNA changed considerably as a result of differential responsiveness to IL-13 stimulation. Such dynamic modulation of isoform expression during stimulation would further tune responsiveness to continuous type 2 cytokine stimulation. Moreover, the differential rate of isoform internalization during cytokine stimulation would also shift the balance of receptors at the plasma membrane available to receive further cytokine stimulation in favor of the defective IL-13Rα1-LOR1a isoform.

Both the expression of the alternative IL-13Rα1-LOR1a receptor isoform per se and its ability to antagonize signaling by the canonical receptor have the potential to interfere with a multitude of biological processes controlled by type 2 cytokines. A role for IL-13Rα1 in the control of serum IgE production has long been established by genetic association and functional studies (10–13) and the B cell–intrinsic role for the IL-13Rα1-LOR1a receptor isoform that we describe here would be consistent with this model. However, although IgE sensitization is a major contributory factor, it may not be sufficient for the development of allergy or atopic disease, which is also influenced by a host of additional genetic and environmental factors. This complex etiology confounds direct associations with IL-13Rα1 expression (14–19). In addition to stochastic allergen exposure, genetic ancestry and biological sex also affect atopic disease susceptibility, through currently unknown mechanisms (14, 15, 18). The direct suppression of *IL13RA1* transcription by female hormones, which we describe here in B cells, may provide a mechanistic explanation for the disparate incidence of IgE sensitization between the sexes (14, 15, 18). The suppressive effect of estrogen is specific to the canonical *IL13RA1* isoform and does not affect the alternative *IL13RA1-LOR1a* isoform. This therefore swings the balance in favor of the *IL13RA1-LOR1a* isoform, via a mechanism that does not appear to involve sexually dimorphic TTP activity. By contrast, *IL13RA1-LOR1a* isoform expression may be influenced by genetic variation independently of biological sex. Indeed, genetic variation in type 2 cytokine, receptor, or adaptor genes, which exhibit different allele frequencies among ethnically diverse populations, are known to affect IgE sensitization and development of allergic disease (5, 46, 57, 58, 62). This notion is supported by an association between *STAT6* polymorphisms previously linked with serum IgE levels (63) and lower *IL13RA1-LOR1a*:*IL13RA1* ratio, as observed here. However, confirmation of this potential association will require larger sample sizes, as well as interrogation of intronic or intergenic SNPs, which may not be in linkage disequilibrium with the exonic SNPs used in this study.

Similar to sex hormone control, the effect of type 2 cytokines or other forms of cellular stimulation on *IL13RA1* transcription differs substantially between distinct cell types. For example, *IL13RA1* transcription is induced by type 2 cytokines in B cells, whereas it is suppressed in monocytes (54). Moreover, type 2 cytokines also suppress certain monocyte responses, particularly type 1 responses to bacterial products (64). In this context, the antagonistic function of the IL-13Rα1-LOR1a isoform would promote the monocyte response. Signaling initiated by IL-13Rα1 in cell types other than B cells may also be relevant for atopic and allergic diseases. For example, IL-13 signaling contributes to asthma through IgE-independent mechanisms (65, 66). Although IL-13Rα2 is not expressed in lymphocytes, it adds another layer of regulation, given its capacity to induce STAT3 phosphorylation after binding IL-13 (67, 68), despite the fact it was originally considered a decoy receptor (5). IL-13Rα1 has also been implicated in additional aspects of B cell homeostasis, such as the expansion of age-associated/autoimmune B cells in lupus (69). Furthermore, type 2 immune signaling is increasingly recognized as a homeostatic mediator of tissue integrity and repair (4) and has, to date, been implicated in diverse responses, including lung (70) and cardiac injury (71), neuroprotection (72), thymic regeneration (73), type 1 diabetes development (74), muscle metabolic fitness (75), intestinal stem cells self-renewal (53), cancer (67, 76–79), and organismal ageing (80). This multifaceted role signifies the need for further understanding of the regulation of type 2 immunity, of which the IL-13Rα1-LOR1a isoform is an important component.

## Materials and Methods

### Study design

This study was designed to dissect the potential function of alternative isoforms of the IL-13Rα1 subunit of the shared type II receptor for IL-4 and IL-13. Using a prior de novo transcriptome assembly and raw RNA-seq data from a variety of sources, we first established the identity and expression pattern of novel *IL13RA1* isoforms, created by exonization of distinct RTEs, and further validated these findings by amplicon sequencing and RT-qPCR assays in human cell lines. The potential function of the most abundant alternative isoform, termed *IL13RA1-LOR1a*, was investigated by knockdown or overexpression in lymphocytic, monocytic and epithelial cell lines, where it was tested for its ability to transmit IL-4 and IL-13 signals. The same experimental settings were also used to test the potential of the alternative IL-13Rα1-LOR1a isoform to interfere with the function of the canonical receptor complex. Finally, the results from experiments in cell lines were validated in similar experiments using primary B cells isolated from the peripheral blood of healthy donors, where responsiveness to IL-13 was correlated with the balance of *IL13RA1* and *IL13RA1-LOR1a* isoform expression. Experiments were repeated independently at least three times and sample sizes and statistical tests are specified in figure legends.

### Transcript identification, read mapping and quantitation

RNA-seq reads, downloaded from The Cancer Genome Atlas TCGA Program (81), GTEx (26), CCLE (48), the EMBL-EBI repository (82), or generated in house were mapped and quantified as previously described (36, 83). Detection of the RTE-exonizing *IL13RA1* isoforms relies on unambiguous mapping of RNA-seq reads to their distinguishing RTE-derived exons. However, as shorter RNA-seq reads from the RTE-derived exons of the alternative *IL13RA1* isoforms are more likely to map also to other genomic locations of the multi-copy RTE families, expression of these RTE-exonizing *IL13RA1* isoforms will be underestimated or become undetectable, as a function of read-length. Annotated gene expression was additionally calculated as previously described (83, 84).

### Repeat annotation

Repeat regions were annotated as previously described (85).

### Sequence alignments

Multiple genomic sequence alignments were carried out using the comparative genomics tool from Ensembl (86). For sequence divergence of the alternative *LOR1a* and canonical *IL13RA1* terminal exons, and splicing and polyadenylation sites, genomic sequences from the following 19 primate species were compared: *Homo sapiens, Pan paniscus, Pan troglodytes, Gorilla gorilla, Pongo abelii, Nomascus leucogenys, Cercocebus atys, Mandrillus leucophaeus, Papio anubis, Macaca fascicularis, Macaca mulatta, Macaca nemestrina, Chlorocebus sabaeus, Rhinopithecus bieti, Rhinopithecus roxellana, Aotus nancymaae, Callithrix jacchus, Cebus capucinus*, and *Saimiri boliviensis boliviensis*.

### Consensus motif identification

Consensus motifs were identified at the *LOR1a* coding sequences, and splicing and polyadenylation sites by sequence alignments using the WebLogo tool (87). The results were plotted as sequence logos.

### Chromatin immunoprecipitation followed by sequencing (ChIP-seq) data

ChIP-Seq data were downloaded from the ReMap (Atlas of Regulatory Regions) track of the UCSC Genome Browser on Human (GRCh38/hg38) (88).

### Functional gene annotation by gene ontology

Pathway analyses were performed using g:Profiler (89) as previously described (83, 84).

### Cell lines

HEK293T (RRID:CVCL_0063), HeLa (RRID:CVCL_0030), SUP-T1 (RRID:CVCL_1714), Jurkat (RRID:CVCL_0065), HARA (RRID:CVCL_2914), LK-2 (RRID:CVCL_1377), HCC4006 (RRID:CVCL_1269), OE19 (RRID:CVCL_1622), PANC-1 (RRID:CVCL_0480), L-1236 (RRID:CVCL_2096), THP-1 (RRID:CVCL_0006), Raji (RRID:CVCL_0511), NCI-H358 (RRID:CVCL_1559), U2OS (RRID:CVCL_0042), NCI-H358 (RRID:CVCL_1559), SQMK-FP (RRID:CVCL_6451), LLC-MK2 (RRID:CVCL_3009), CP132 (RRID:CVCL_4148) and CM0203F (90), were obtained from the Cell Services facility of The Francis Crick Institute and verified as mycoplasma-free. All human cell lines were further validated by DNA fingerprinting. Cells were grown in IMDM, DMEM, or RPMI, with 5% or 10% heat-inactivated fetal calf serum (FCS) (Gibco), as indicated in table S1. All media were supplemented with L-glutamine (2 mM) (Thermo Fisher Scientific) and penicillin–streptomycin (50 U/ml) (Gibco, Cat #15070063). Recombinant IL-4 (Peprotech, Cat #200-04) and IL-13 (Peprotech, Cat #200-13) were added at 20 ng/ml unless otherwise specified. 17β-estradiol (Sigma-Aldrich, Cat #E8875) was dissolved in 99% ethanol prior to treatment at the concentrations indicated in fig S5B.

### Primary B cells

Donor peripheral blood mononuclear cells were purchased from TebuBio, UK or NHSBT, UK, then processed with Lymphoprep (STEMCELL, Cat #07851) according to manufacturer’s instructions and prepared for storage in liquid N_2_. B cells were later isolated from thawed vials by negative isolation (STEMCELL, Cat #17954), prior to resuspension in RPMI supplemented with heat-inactivated fetal bovine serum (Gibco), L-glutamine (2 mM) (Thermo Fisher Scientific), and penicillin–streptomycin (50 U/ml) (Gibco, Cat #15070063). Ethics approval was obtained from the Research Ethics Committee (REC reference: 20/PR/0400).

### Expression vector transfections and transductions

pcDNA3 Myc2-Hs TTP WT for overexpression of *ZFP36* was a gift from J. Downward’s lab at the Francis Crick Institute (91). The spliced coding sequences for canonical *IL13RA1, IL13RA1-AluJb*, and *IL13RA1-LOR1a* were synthesized and inserted into the pRV-IRES-GFP or pRV-IRES-TdTomato mammalian retroviral production vectors. Site-directed mutagenesis (SDM) was performed to mutate the two tyrosine-encoding sites in canonical *IL13RA1* to phenylalanines. Viral supernatant was harvested 72 hours after HEK293T cells were cotransfected with associated pRV vector along with pVSV-G and pHIT60 retroviral production vectors using GeneJuice transfection reagent (Sigma-Aldrich, Cat #70967), and target cells were transduced by centrifugation in the presence of viral supernatant and polybrene (4 μg/ml) at 300*g* for 45 min at room temperature. Canonical *IL4RA* was synthesized and inserted into pcDNA3.1, and SDM altered its six encoded tyrosines to phenylalanines. IL4RA and mIL4RA were transfected using GeneJuice transfection reagent (Sigma-Aldrich, Cat #70967) for 48 hours. Genscript performed gene synthesis, cloning and SDM, and plasmids were verified by whole plasmid sequencing by Plasmidsaurus using Oxford Nanopore Technology with custom analysis and annotation.

### CRISPR/Cas9-mediated editing

Deletion of the *LOR1a* element splice acceptor site downstream of the *IL13RA1* gene was achieved by single guide RNAs (sgRNAs) targeting sequences (table S3) either side of it. These were designed using the CRISPOR online tool (92). Editing was carried out by transfection of sgRNA and recombinant Cas9 protein (Thermo Fisher Scientific, Cat #A36498) using Lipofectamine CRISPRMAX Cas9 Transfection Reagent (Thermo Fisher Scientific, Cat #CMAX00003) according to manufacturer’s instructions. Cells were seeded 1 day prior to transfection with the pool of guides (in pairs straddling the *LOR1a* element splice acceptor site). Two days after transfection, cells were treated with IL-13. RNA was then isolated after a further 2 days.

### Knockdown via oligonucleotides

Knockdown of *IL13RA1-LOR1a* was achieved by transfection of ASOs with a phosphorothioate modification applied to every base and Affinity Plus™ modifications to the first three 5′ and last three 3′ bases, targeting the *IL13RA1-LOR1a* splice junction (ASO_splice) or the *LOR1a* exon (ASO_exon) or using Dicer-Substrate siRNA targeting the *LOR1a* element (93). Oligonucleotide sequences are provided in table S3. Transfection was achieved using Lipofectamine RNAiMAX transfection reagent (Thermofisher, Cat #13778100) according to manufacturer’s instructions, with 5 pmol of oligonucleotide per well in 24-well plates.

### RNA preparation, PCR, and sequencing

For bulk RNA sequencing of Raji cells, RNA was extracted from cell lines using RNeasy kit (Qiagen, Cat #74104) and library prep was performed with NEBNext Ultra II Directional PolyA mRNA kit (NEB, Cat #E7760). Samples were then sequenced on a NovaSeq (Illumina). For all other assays, RNA was extracted from cells using RNeasy kit (Qiagen, Cat #74106), and subsequent cDNA synthesis was performed using High-Capacity Reverse Transcription Kit (Thermofisher, Cat #4368813). Gene-, isoform-, and species-specific primers (table S2) were designed to quantify expression in cDNA using quantitative PCR using Quantstudio 3 (Applied Biosystems) and Fast SYBR Green master mix (Thermofisher, Cat #4385617). The Dreamtaq polymerase (Thermofisher, Cat #EP0701) kit was used for end-point PCR to generate samples for Sanger sequencing, which was performed by Genewiz, Essex, UK, using the same primers. Unless otherwise specified, primers were designed for qPCR and against the human genome, and values were normalized to *HPRT1* expression using the ΔC_T_ method.

### Protein structure prediction and visualization

Structures of the canonical IL-13Rα1 and alternative isoforms were modeled based on the structure of the canonical IL-13Rα1 (ID: AF-P78552-F1) predicted by AlphaFold (94). Structures were visualized in the Research Collaboratory for Structural Bioinformatics Protein Data Bank Mol* 3D Viewer (95).

### Protein immunoblotting

Cell samples were lysed in RIPA buffer (SigmaAldrich, Cat #R0278) supplemented with protease inhibitor (Thermo, Cat #1862209), phosphatase inhibitor (Thermo, Cat #1862495), 0.2 mM PMSF (Thermo, Cat #36978), and 5 mM EDTA (Thermo, Cat #1861274), then denatured for 10 min at 95°C in LDS sample buffer (Invitrogen, Cat #2463558) supplemented with 1M DTT (Invitrogen, Cat #P2325), run on a 4 to 20% SDS Precast Protein Gel (BioRad, Cat #4561094) and transferred using Turbo transfer system (BioRad, Cat #1704150) with a Trans Blot Turbo Mini 0.2-μm Nitrocellulase Transfer Pack (BioRad, Cat #1704158). Membranes were washed in TBS-T and blocked for 30 min in 5% (w/v) milk in TBS-T. Membranes were incubated with primary antibodies rabbit anti-pSTAT6 (1:1000, Cell Signaling Technology, Cat #9361) and rabbit anti- pan-actin (1:5000, Cell Signaling Technology, Cat #8456) overnight. Membranes were then washed five times in TBS-T prior to 1 hour incubation with a secondary antibody (anti-rabbit HRP (1:5000) Cell Signaling Technology, Cat #7074)). Membranes were again washed five times in TBS-T prior to incubation with Western ECL Substrate (BioRad, Cat #1705060) and imaging on Amersham Imager 600 (GE Healthcare). Densitometry of band intensity was calculated using ImageJ image analysis software, version 2.14.0/1.54f. Uncropped blots are provided in data file S2.

### Flow cytometry and cell sorting

Cell suspensions were stained for 30 min with the following anti-human antibodies from BioLegend: PE-conjugated anti-IL-13Rα1 (1:100) (Cat #360404), APC-conjugated anti-IL-13Rα1 (1:100) (Cat #360406), APC-conjugated anti-IL-4Rα (1:200) (Cat #355005), Brilliant discrimination. Violet™ 421–conjugated anti-CD23 (1:100) (Cat #338522), APC-conjugated anti-CD19 (1:200) (Cat #302212), and PE-conjugated anti-STAT6 phospho (Tyr641) (1:50) (Cat #686004). One hundred nanomolar DAPI (Sigma-Aldrich, Cat #10236276001) was used for any live-cell discrimination.

Samples were run on a BD Biosciences LSR Fortessa using FACSDiva v8.0 and analyzed with FlowJo v10. Gating strategies for individual cell lines are provided in fig. S18. Cell sorting was performed using either a FACSAria Fusion cell sorter (BD) or an Avalon cell sorting system (Bio-Rad). For primary samples, cell suspensions were fixed then permeabilized (eBioscience, Cat #88-8824-00), prior to incubation with staining solution of antibodies in permeabilization buffer (eBioscience, Cat #88-8824-00). Cells were washed with 1% (w/v) BSA in PBS. Cells were also resuspended in 1% (w/v) BSA in PBS prior to acquisition.

### Image flow cytometry

Image flow cytometry was performed on a Cytek® Amnis® ImageStream®^X^ Mk II Imaging Flow Cytometer. Focus was maintained by automated software through contiguous running of Amnis® SpeedBead® kit beads (Amnis®, Cat #CN-0440-01). Capture was performed at a pixel size of 0.3 × 0.3 μm at 60X magnification with an aperture of 0.9, a field of view of 40 × 170 μm and at a maximum imaging rate of 1200 cells per second. Cells were imaged live and suspended in room temperature 1% (w/v) BSA in PBS. Illumination was achieved with a four-laser set: 488 nm, 561 nm, 405 nm, and 640 nm in the CellStream® optical layout using Cytek®’s patented time delay integration and camera technology. Machine channel 3 (560-595 nm) was activated for capture of PE fluorescence, machine channel 7 (420-505 nm) for DAPI, and machine channel 11 (595-660 nm) for APC. Images were acquired with Cytek® INSPIRE™ software v201.2.0.7. A compensation file was generated using single-color controls and applied during analysis using IDEAS® v6.4 software, and cells were gated on live cells, single cells, in focus, and positive for both CD45RA and IL-13Rα1 staining. Internalization ratios were determined using the built-in internalization analysis wizard, using the CD45RA channel as a surface marker for masking. Briefly, the ratio represented the intensity of the APC IL-13Rα1 channel within the confines of the PE CD45R surface mask, compared to what overlapped it. Cell suspensions were stained for 30 min with the following anti-human antibodies from BioLegend: APC-conjugated anti-IL-13Rα1 (1:50) (Cat #360406) and PE-conjugated anti-CD45RA (1:200) (Cat #304108). Cells were then washed and incubated at 37°C in 1% (w/v) BSA in PBS for indicated time points.

### Statistical analyses

Statistical comparisons were made using GraphPad Prism 10 (GraphPad Software) or SigmaPlot 14.0. Parametric comparisons of normally distributed values that satisfied the variance criteria were made by unpaired or paired two-way Student’s *t* tests, one-way analysis of variance (ANOVA) tests with Bonferroni or Šidák correction for multiple comparisons, one-way repeated measures ANOVA tests with Bonferroni or Tukey correction for multiple comparisons, or two-way repeated measures ANOVA test with Šidák correction for multiple comparisons. Data that did not pass the variance test were compared with nonparametric two-tailed Mann–Whitney *U* tests (for unpaired comparisons), Wilcoxon signed-rank tests (for paired comparisons), or ANOVA on ranks tests with Tukey or Dunn correction for multiple comparisons.

## Supplementary Material

data file S1

data file s2

data file s3

Supplementary Material

## Data Availability

The RNA-seq data generated in this study have been deposited at the EMBL-EBI repository (82) (E-MTAB-14186). Other publicly available dataset supporting the findings of this study included the following: GTEx mRNA expression data from healthy tissues (26); microarray data from LCLs from healthy individuals of diverse ancestry (GSE12526) and from children from families recruited through a proband with asthma (E-MTAB-1425) (35); ChIP-Seq data from the ReMap (Atlas of Regulatory Regions) track of the UCSC Genome Browser on Human (GRCh38/hg38) (88); RNA-seq data from the Cancer Cell Line Encyclopedia (CCLE) (48); RNA-seq data from LCLs from healthy individuals of diverse ancestry (PRJEB3366) sequenced as part of the 1000 Genomes Project; RNA-seq data from isolated peripheral blood cell populations from healthy donors and patients with type 1 diabetes, multiple sclerosis, or amyotrophic lateral sclerosis (SRP045500) (39); RNA-seq data from healthy donor PBMCs over the course of IAV vaccination (E-GEOD-45735) (44); RNA-seq data from B cells isolated from adolescents with or without IgE-mediated food allergies (GSE165316) (47); RNA-seq data from healthy donor PBMCs across the age spectrum (GSE193141) (49); full-length RNA-seq data from healthy human PBMCs (SRR9944890) (38); and full-length RNA-seq data from healthy human PBMCs (37). Tabulated data underlying Figs. 1 to 6 and figs. S1, S4 to S13, and S15 to S17 are archived in data file S3. All other data needed to support the conclusions of the paper are present in the main text or supplementary materials. For the purpose of open access, the author has applied a CC BY-ND public copyright license to any author accepted manuscript version arising from this submission.

## One-sentence summary

Co-option of primate-specific retroelements creates an alternative IL- 13Rα1 subunit that, together with sexually dimorphic IL-13Rα1 expression, tune type 2 immune responses.
