# Supplementary figures and images for: Primate retroelement exonization and sexually dimorphic IL13RA1 transcription tune type 2 immune responses
Source: Sci Immunol. Author manuscript; Available in PMC 2025 Aug 7. (PMC7617988; doi:10.1126/sciimmunol.adr1105)

Unmodified/uncropped blots

Fig. 3B pSTAT6

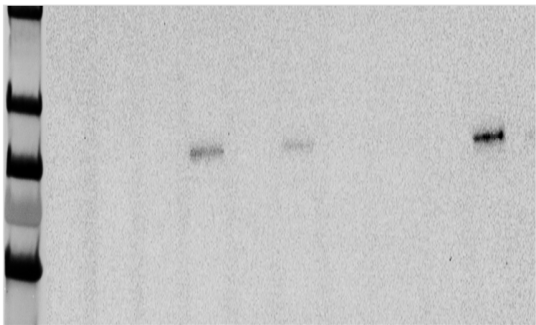

Fig. 3B Actin

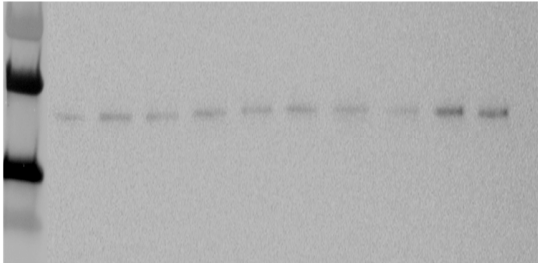

Fig. S4A

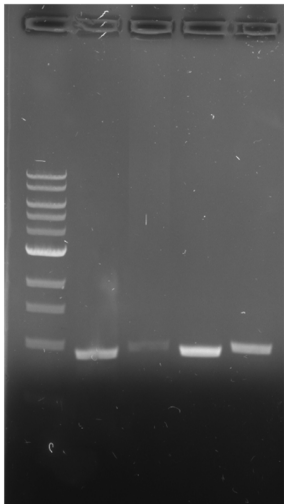

Fig. S4B

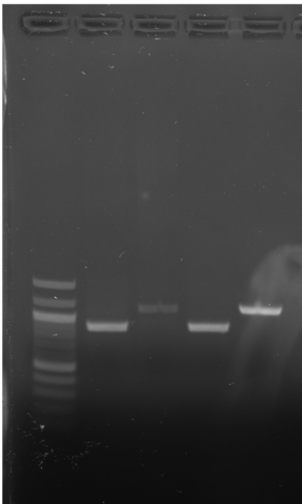

Fig. S6B pSTAT6

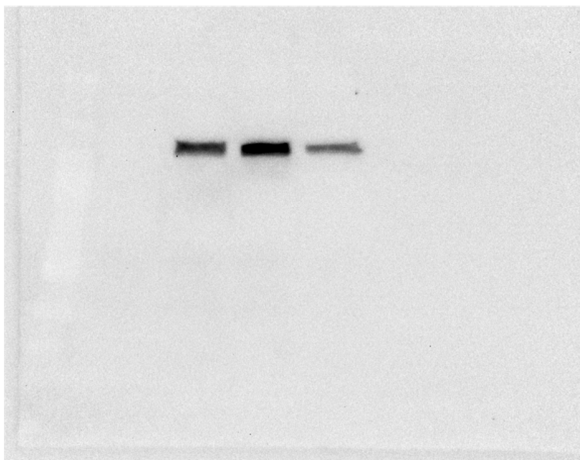

Fig. S6B Actin

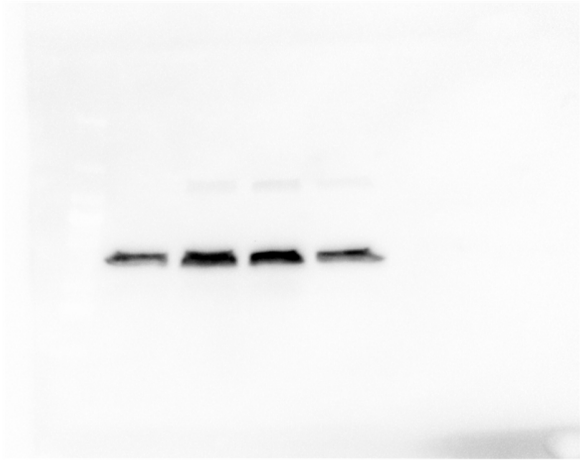

Supplement: data file s2 [file EMS207658-supplement-data_file_s2.pdf]
