# Supplementary material for: Primate retroelement exonization and sexually dimorphic IL13RA1 transcription tune type 2 immune responses
Source: Sci Immunol. Author manuscript; Available in PMC 2025 Aug 7. (PMC7617988; doi:10.1126/sciimmunol.adr1105)
Supplement: Supplementary Material [file EMS207658-supplement-Supplementary_Material.pdf]

Supplementary Materials for

Primate retroelement exonization and sexually dimorphic *IL13RA1* transcription tune type 2 immune responsesRetroelement exonization by primate *IL13RA1* tunes type 2 immune responses

Tobias Plowman, Tom Hofland, Callum Hall, Rachael Thompson, Judith Pape, Kevin W Ng, Laura Doglio, and George Kassiotis

Corresponding author: george.kassiotis@crick.ac.uk

The PDF file includes:

Figs. S1 to S18
Tables S1 to S3
Captions for data files S1 to S3

Other Supplementary Material for this manuscript includes the following:

Data files S1 to S3
MDAR Reproducibility Checklist

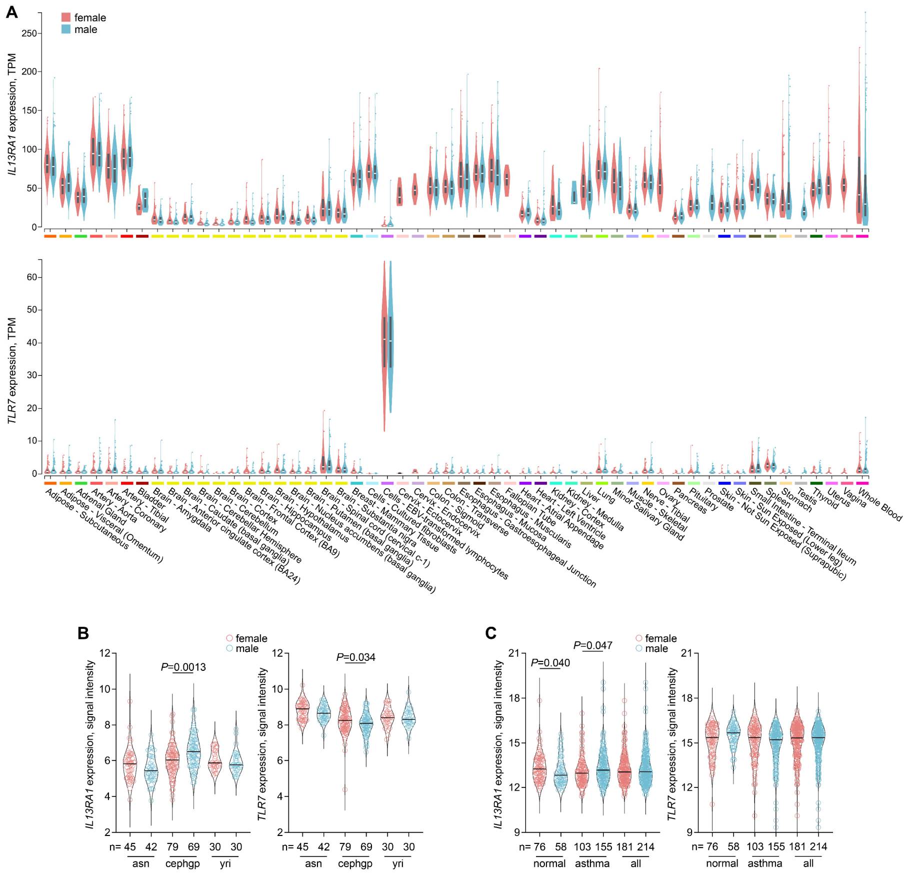

Fig. S1. Sexual dimorphism in *IL13RA1* expression. (**A**) Expression (TPM) of *IL13RA1* and *TLR7* in the indicated tissues from male and female donors (*n* = 10-541). Data were downloaded from the GTEx Portal (*26*). (**B**) Expression (signal intensity) of *IL13RA1* and *TLR7* in microarray data (GSE12526) from male and female donors of the indicated ancestry (asn, Asian; cephgp; European; yri: African). (**C**) Expression (signal intensity) of *IL13RA1* and *TLR7* in microarray data (E-MTAB-1425) from male and female children from families recruited through a proband with asthma. In (B and C), number of independent donors are indicated in the figure and *P* values were calculated with two-tailed Student's *t* tests comparing males and females.

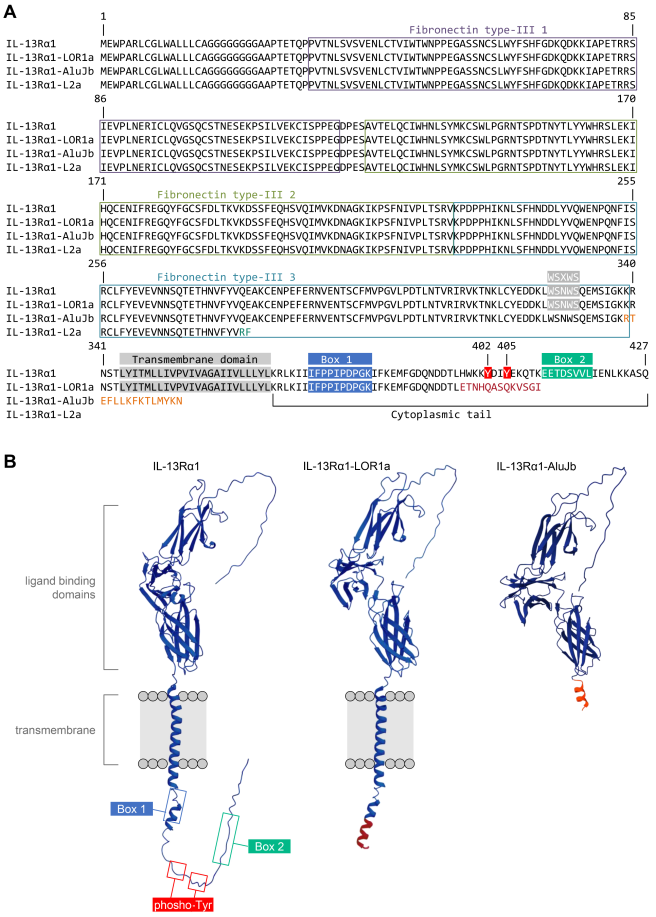

Fig. S2. Protein sequence and predicted structure of IL-13Rα1 isoforms. (**A**) Amino acid sequence alignment of the indicated IL-13Rα1 isoforms. Also indicated are the three Fibronectin type-III domains and WSXWS motif in the extracellular part, the transmembrane domain, and the Box 1, phospho-tyrosines and Box 2 in the cytoplasmic tail. (**B**) AlphaFold-predicted structure of the indicated IL-13Rα1 isoforms. Canonical IL-13Rα1 sequences and sequences translated from exonised RTEs are shown in blue and red colors, respectively.

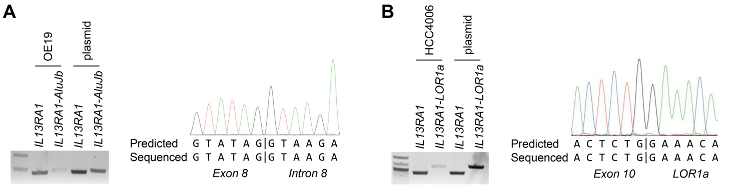

Fig. S3. Sequencing confirmation of novel *IL13RA1-AluJb* and *IL13RA1-LOR1a* junctions. (**A**) Left: PCR amplicons corresponding to the *IL13RA1* and *IL13RA1-AluJb* isoforms in OE19 cells. Amplicons obtained using plasmid DNA templates for each isoform are also included as controls. Right: part of the *IL13RA1-AluJb* amplicon sequencing chromatogram from OE19 cells, at the junction between exon 8 and intron 8. (**B**) Left: PCR amplicons corresponding to the *IL13RA1* and *IL13RA1-LOR1a* isoforms in HCC4006 cells. Amplicons obtained using plasmid DNA templates for each isoform are also included as controls. Right: part of the *IL13RA1-LOR1a* amplicon sequencing chromatogram from HCC4006 cells, at the junction between exon 10 and the downstream *LOR1a* element.

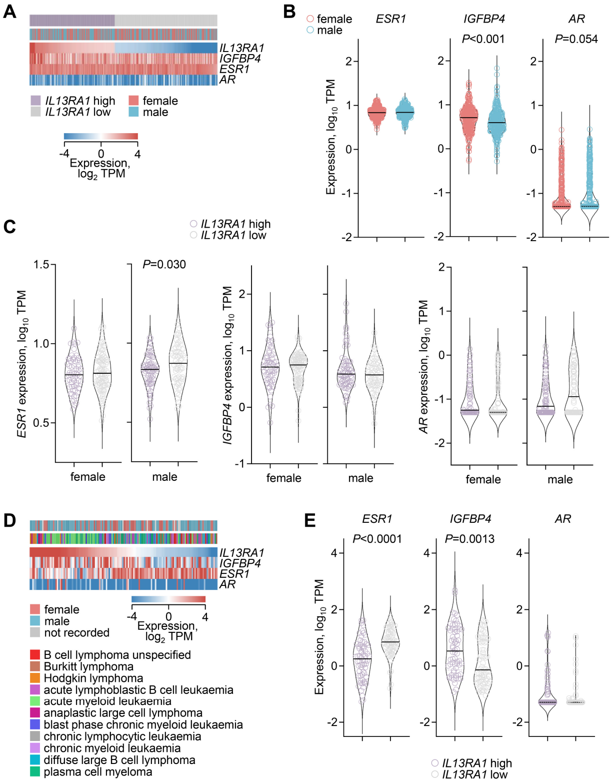

Fig. S4. Estrogen control of *IL13RA1* expression. (**A** to **C**) Expression of the indicated genes in RNA-seq data from LCLs from healthy individuals (PRJEB3366). In A, samples with high (≥ 2 TPM, n = 131) and low (≤ 0.4 TPM, n = 158) *IL13RA1* expression are compared, and in (C), the same cut-offs are used for female (n = 59 and n = 93) and male donors (n = 72 and n = 65) for high and low *IL13RA1* expression, respectively. In (B), all female (n = 337) and male donors (n = 329) are compared. The correlation between *IL13RA1* expression and *IGFBP4* expression is significant ($P < 0.001$, q < 0.001, linear regression R = 0.482). (**D** to **E**) Expression of the indicated genes in RNA-seq data from CCLE hematopoietic cell lines with high (≥ 2 TPM, n = 64) and low (≤ 0.4 TPM, n = 74) *IL13RA1* expression. In (B, C and E), P values were calculated with two-tailed Student's t tests (C, E *ESR1*) or Mann–Whitney U tests (B, E *IGFBP4*).

Commented [SS1]: Which tests for which parts?

Commented [GK2R1]: These are now specified

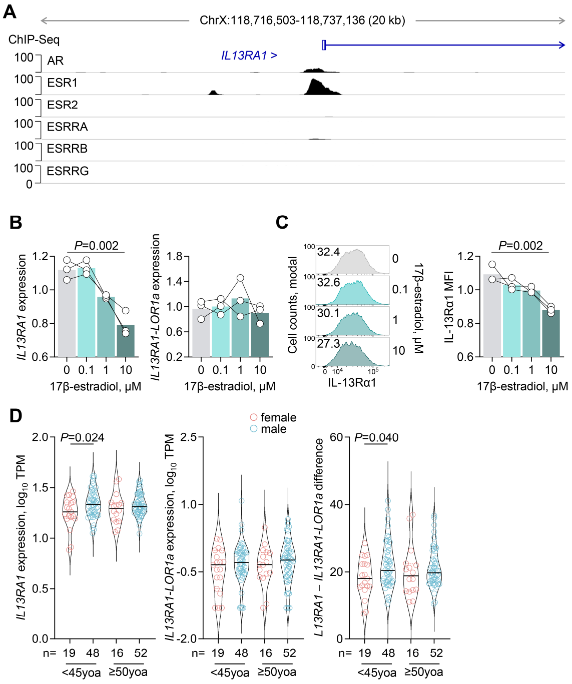

Fig. S5. Estrogen control of *IL13RA1* expression. (**A**) Binding of the indicated transcription factor in the *IL13RA1* promoter region in chromatin immunoprecipitation followed by sequencing (ChIP-seq) data downloaded from the ReMap (Atlas of Regulatory Regions) ChIP-seq track of the UCSC Genome Browser GRCh38/hg38 (*88*). (**B** and **C**) (B) *IL13RA1* and *IL13RA1-LOR1a* mRNA and (C) IL-13Rα1 protein expression in the Hodgkin lymphoma L-1236 cell line following treatment with the indicated doses of 17β-estradiol. *IL13RA1* and *IL13RA1-LOR1a* mRNA expression was determined by RT-qPCR and *HPRT1* expression-normalized values are plotted relative to those of untreated control cells in each replicate. IL-13Rα1 protein expression was determined by flow cytometry, as exemplified in (C) (left) and numbers within the plots denote the MFI $\times 10^{-3}$. In (C) (right), the MFIs of IL-13Rα1 staining in treated cells are plotted relative to those of untreated control cells in each replicate. In (B and C) (right), symbols represent the pooled independent experiments ($N = 3$), each connected with lines and P values were calculated with a one-way repeated measures ANOVA test and Tukey correction for multiple comparisons, with the untreated group as control. (**D**) Expression of *IL13RA1* and *IL13RA1-LOR1a*, and their difference, in RNA-seq data from healthy donor PBMCs (GSE193141), according to donor sex and age. Number of independent donors are indicated in the figure and P values were calculated with two-tailed Student's t tests between male and female donors of the same age group.

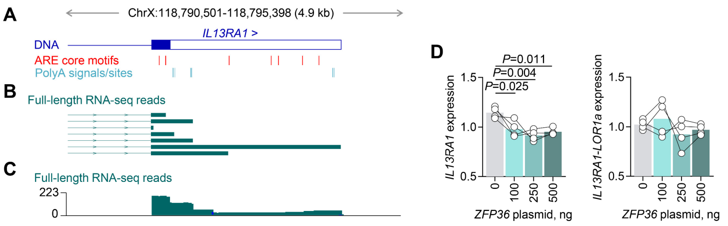

Fig. S6. *ZFP36*-mediated control of *IL13RA1* expression. (**A**) Location of ARE core motifs (ATTTA) and polyadenylation (polyA) signals ((A/T)(A/T)TAAA)) and sites (CA) in the canonical *IL13RA1* 3′ UTR. (**B**) Collapsed transcripts built from full-length RNA-seq reads from healthy human PBMCs (PacBio Multiplexed Arrays Sequencing) in the IL13RA1 locus. (**C**) Trace of full-length RNA-seq reads in data from healthy human PBMCs (SRR9944890). The data in (B and C) are from Fig. 1, B and C, respectively, zoomed in on the canonical *IL13RA1* 3′ UTR. (**D**) *IL13RA1* and *IL13RA1-LOR1a* expression, determined by RT-qPCR and plotted relative to *HPRT1* expression, in LK-2 cells transfected with the indicated amount of a *ZFP36*-expressing plasmid. Symbols represent ~~independent~~ the pooled experiments (*N* = 4), each connected with lines and *P* values were calculated with a one-way repeated measures ANOVA test and Tukey correction for multiple comparisons, with the untransfected group as control.

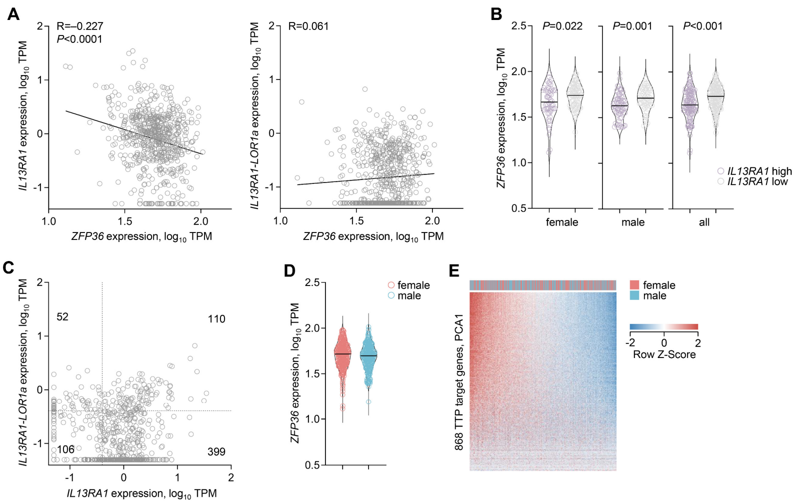

Fig. S7. *ZFP36*-mediated control of *IL13RA1* expression. (**A**) Correlation of *ZFP36* and either *IL13RA1* (left) or *IL13RA1-LOR1a* expression (right) in RNA-seq data from LCLs ($n = 666$) from healthy individuals (PRJEB3366). (**B**) *ZFP36* expression in LCLs from female ($n = 59$ and $n = 93$), male ($n = 72$ and $n = 65$) and all donors ($n = 131$ and $n = 158$) with high (≥ 2 TPM) and low (≤ 0.4 TPM) *IL13RA1* expression, respectively. *P* values were calculated with two-tailed Student's *t* test~~s~~ (male) or Mann–Whitney *U* tests (female, all). (**C**) Correlation of *IL13RA1* and *IL13RA1-LOR1a* expression in RNA-seq data from the same LCLs. Dashed lines represent the cut-offs (≤ 0.4 TPM) used to define LCLs with low expression of each isoform. (**D**) Expression of *ZFP36* in RNA-seq data from female ($n = 337$) and male ($n = 330$) LCLs from the same dataset. (**E**) Heatmap of expression of 868 TTP target genes plotted according to principal component analysis 1 (PCA1) in the same LCLs. Donor sex is also indicated.

Commented [SS3]: Specify

Commented [GK4R3]: These too are now specified

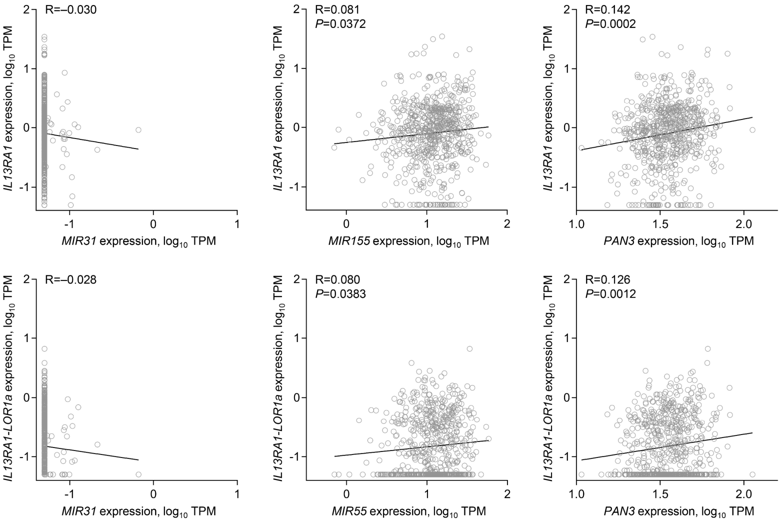

Fig. S8. Possible effect of *MIR31*, *MIR155* and *PAN3* on *IL13RA1* expression. (**A**) Correlation of *IL13RA1* (left) or *IL13RA1-LOR1a* expression (right) with expression of *MIR31* (top), *MIR155* (middle) or *PAN3* (bottom) in RNA-seq data from LCLs (n = 667) from healthy individuals (PRJEB3366).

A

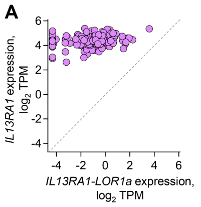

B

Gene	SNP ID	hg38 position	Reference allele	Europeans	Africans	Asians
IL13RA1	rs2495636	chrX:118791935	A	0.839	0.904	0.630
IL13RA1-LOR1a	rs2495639	chrX:118804965	G	0.716	0.622	0.586
IL13	rs20541	chr5:132660272	A	0.197	0.195	0.304
IL4	rs2070874	chr5:132674018	C	0.856	0.599	0.219
IL4RA	rs1801275	chr16:27363079	A	0.797	0.329	0.822
STAT6	rs4559	chr12:57095865	C	0.349	0.478	0.712

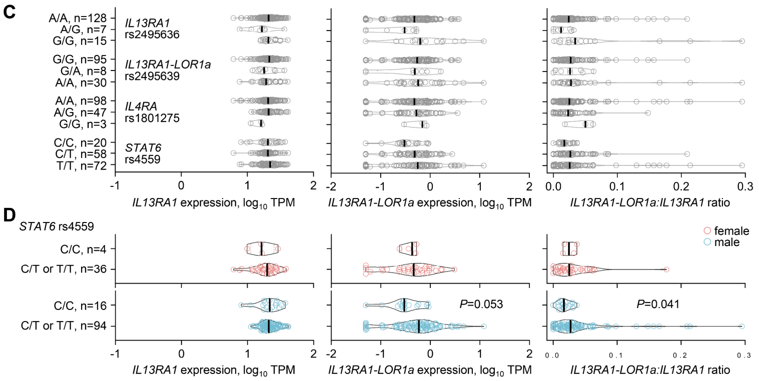

Fig. S9. Effect of genetic variation in type 2 cytokine, receptor and adaptor genes on the balance of *IL13RA1* isoform expression. (**A**) Correlation of *IL13RA1* and *IL13RA1-LOR1a* expression in RNA-seq data from healthy donor PBMCs (GSE193141). ~~Each symbol is an individual donor (*n* = 150).~~ (**B**) Chromosomal position and frequencies of the reference alleles in populations of different genetic ancestry of selected SNPs in transcribed regions of the indicated genes. Data were obtained from the National Center for Biotechnology Information (NCBI) SNP database (*59*). (**C**) *IL13RA1* and *IL13RA1-LOR1a* expression, and ratio of *IL13RA1-LOR1a*:*IL13RA1* expression in the same individuals as in A, according to inferred genotype at the indicated loci, based on RNA-seq data. (**D**) *IL13RA1* and *IL13RA1-LOR1a* expression, and ratio of *IL13RA1-LOR1a*:*IL13RA1* expression in the same individuals as in (A), stratified by homozygosity for the reference allele at the *STAT6* rs4559 SNP and donor sex. In (A, C and D), symbols represent individual donors (*n* = 150). *P* values were calculated with Mann–Whitney *U* tests.

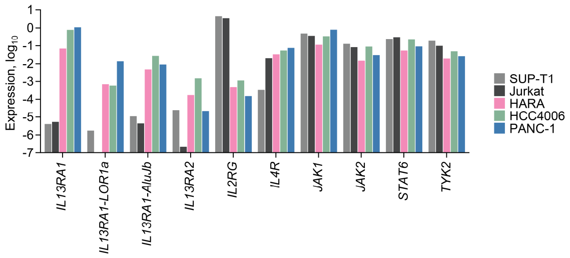

Fig. S10. Expression of *IL13RA1* and related receptor subunits and signaling adaptors in cell lines. ~~The average e~~Expression of the indicated gene, determined by RT-qPCR and plotted relative to *HPRT1* expression, in the indicated cell lines is shown (*N* = 1). ~~A single experiment was performed.~~

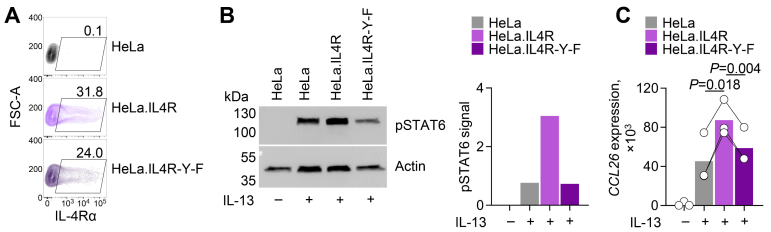

Fig. S11. Tyrosine phosphorylation in IL-4Rα subunit signaling. (**A**) Expression of the IL-4Rα subunit, determined by flow cytometry, in parental HeLa cells or HeLa cells transfected to express wild-type IL-4Rα (HeLa.IL4R) or an IL-4Rα mutant with all six tyrosines in the intracellular tail replaced with phenylalanines (HeLa.IL4R-Y-F) (*N* = 1). (**B**) Left: protein immunoblot of phosphorylated STAT6 (pSTAT6) in the same cell lines after treatment with IL-13. Untreated HeLa cells are also included as controls. Right: densitometric quantitation of pSTAT6 signals, according to the loading control (actin). Results are from a single experiment (*N* = 1). (**C**) Expression of *CCL26*, determined by RT-qPCR and plotted relative to *HPRT1* expression, in the same cell lines as in (B). Symbols represent the pooled independent experiments (*N* = 3), each connected with lines. *P* values were calculated with a one-way repeated measures ANOVA test and Tukey correction for multiple comparisons for the treated groups.

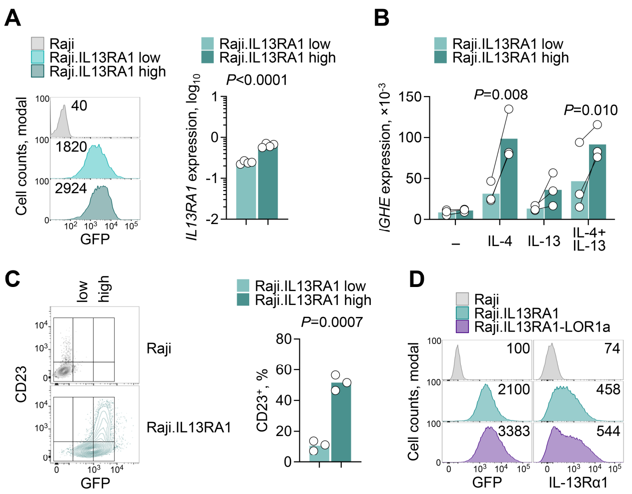

Fig. S12. Sensitivity of type 2 cytokine signaling to IL-13Rα1 levels. (**A**) Left: representative flow cytometry plots of the GFP reporter for IL-13Rα1 expression in parental Raji cells or Raji cells transduced to express different IL-13Rα1 levels (Raji.IL13RA1 low or high). Numbers denote the median fluorescence intensity (MFI) of GFP. Right: *IL13RA1* expression, determined by RT-qPCR and plotted relative to *HPRT1* expression, in the same cell lines. Symbols represent the pooled independent ~~measurements~~ experiments ~~-~~(~~*n*~~ *N* = 4) and the *P* value was calculated with a two-tailed Student's *t* test. (**B**) *IGHE* expression, determined by RT-qPCR and plotted relative to *HPRT1* expression, in the same cell lines prior and following treatment with IL-4, IL-13, or both. Symbols represent ~~independent~~ the pooled experiments (~~*n*~~ *N* = 3), each connected with lines. *P* values were calculated with a two-way repeated measures ANOVA test and Šídák correction for multiple comparisons. (**C**) Left: representative flow cytometry plots of CD23 staining, according to GFP expression ~~in the same cell lines as in~~ in parental Raji cells and Raji.IL13RA1 low from (A) and (B)~~(A)~~, following treatment with IL-13. Right: percentage of CD23$^+$ cells in ~~the same cells~~the indicated GFP gates. Symbols represent independent the pooled independent experiments (~~*n*~~ *N* = 3) and the *P* value was calculated with a paired two-tailed Student's *t* test. (**D**) Representative flow cytometry plots of IL-13Rα1 and GFP reporter expression in parental Raji cells or Raji cells transduced to express IL-13Rα1 (Raji.IL13RA1) or IL-13Rα1-LOR1a (Raji.IL13RA1-LOR1a) and GFP. Numbers denote the MFI.

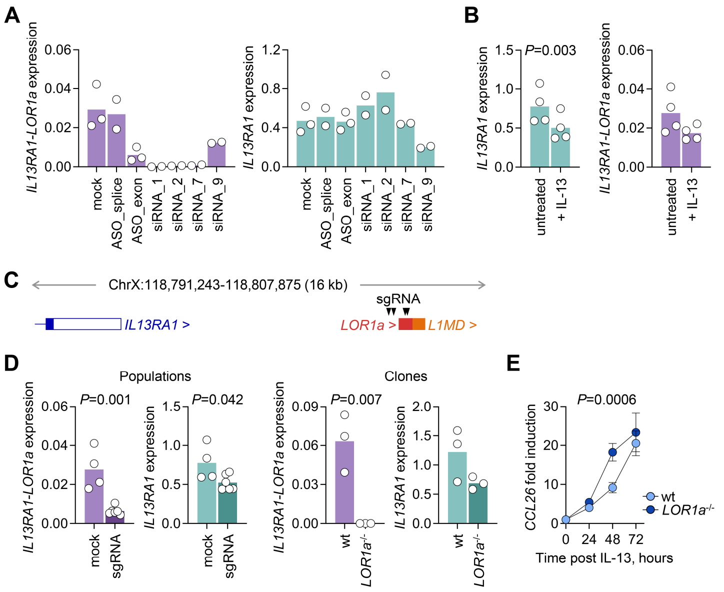

Fig. S13. Effect of knockdown or CRISPR/Cas9-mediated mutation of the *LOR1a* element. (**A**) *IL13RA1* and *IL13RA1-LOR1a* expression, determined by RT-qPCR and plotted relative to *HPRT1* expression, in U2OS cells that were either mock-transfected or transfected with ASOs targeting the *IL13RA1-LOR1a* splice junction (ASO_splice) or the *LOR1a* exon (ASO_exon), or with siRNA oligonucleotides targeting the *LOR1a* element. (**B**) *IL13RA1* and *IL13RA1-LOR1a* expression, determined by RT-qPCR and plotted relative to *HPRT1* expression, in mock-transfected NCI-H358 cells before and after IL-13 treatment. (**C**) Position of sgRNAs (black arrows) targeting the *LOR1a* element splice acceptor site. (**D**) *IL13RA1* and *IL13RA1-LOR1a* expression, determined by RT-qPCR and plotted relative to *HPRT1* expression, in bulk populations (left) or single-cell clones (right) in NCI-H358 that were mock-transfected (without sgRNA or with a non-targeting sgRNA) (mock and wt, respectively) or transfected with *LOR1a*-targeting sgRNA, used in pairs straddling the *LOR1a* element splice acceptor site (sgRNA and *LOR1a*$^{~~+/-~~ -/-}$, respectively). (**E**) Mean ± SEM (~~*n*~~ *N* = 3) *CCL26* fold-induction over the course of IL-13 treatment in wt and *LOR1a*$^{~~+/-~~ -/-}$ NCI-H358 clones. ~~In~~ (A, B and D) S~~, s~~ymbols represent the pooled ~~individual replicates from three~~ independent experiments (*N* = 2 to 4). ~~and~~ *P* values were calculated with (B) paired two-tailed Student's *t* test, (D) unpaired two-tailed Student's *t* tests, or (E) Area under dose-response. The same control cells were used in (B) ~~(~~untreated~~)~~ and (D) ~~(~~mock~~)~~.

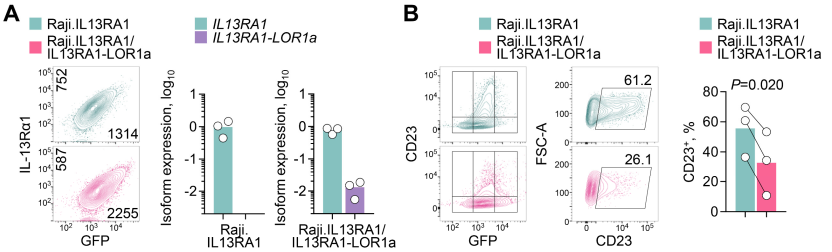

Fig. S14. IL-13Rα1-LOR1a antagonism of IL-13Rα1 signaling. (**A**) Left: representative flow cytometry plots of IL-13Rα1 and GFP reporter expression in Raji cells transduced to express only IL-13Rα1 (Raji.IL13RA1) or both IL-13Rα1 and IL-13Rα1-LOR1a (Raji.IL13RA1/IL13RA1-LOR1a) and GFP. Numbers denote the MFI of IL-13Rα1 and GFP, on the respective axes. Right: *IL13RA1* and *IL13RA1-LOR1a* expression, determined by RT-qPCR and plotted relative to *HPRT1* expression, in the same cells. Symbols represent the pooled independent ~~measurements~~experiments (N = 3). (**B**) Left: representative flow cytometry plots of ~~–~~ CD23 staining, according to GFP expression, in the same cell lines as in (A) following treatment with IL-13. Right: percentage of $CD23^+$ cells in the same cells gated for intermediate GFP expression. Symbols represent the pooled independent experiments (~~n~~ N = 3). ~~and the~~The P value was calculated with a two-tailed paired Student's t test.

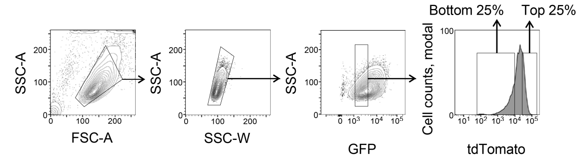

Fig. S15. FACS strategy for isolating Raji.IL13RA1/IL13RA1-LOR1a cells. Representative gating strategy to isolate Raji.IL13RA1/IL13RA1-LOR1a cells expressing intermediate GFP levels. The bottom and top quartiles of tdTomato levels before or after IL-13 treatment are also shown.

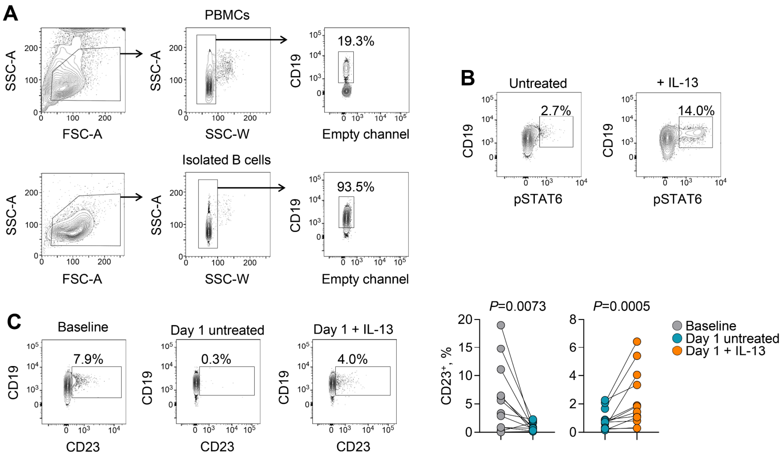

Fig. S16. Primary B cell flow cytometry gating strategies. (**A**) Example of gating for the identification of B cells (CD19+ cells in PBMCs) before (top) and after B cell isolation (bottom). (**B**) Example of gating for detection of pSTAT6 expression in isolated B cells with or without IL-13 treatment. (**C**) Example of gating for detection of CD23 expression (~~top~~left) and percentage of CD23+ cells directly after B cell isolation and after 1 day of culture with or without IL-13 treatment (right). Symbols represent individual donors (n = 17).

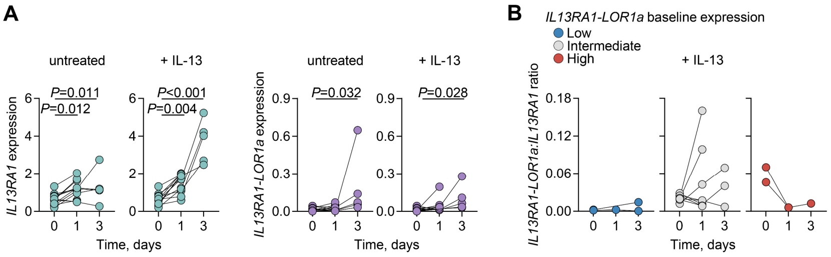

Fig. S17. Modulation of *IL13RA1* and *IL13RA1-LOR1a* expression by IL-13 stimulation. (**A**) *IL13RA1* (left) and *IL13RA1-LOR1a* expression (right), determined by RT-qPCR and plotted relative to HPRT1 expression, in peripheral blood primary B cells over the course of in vitro culture with (+ IL-13) or without (untreated) IL-13 stimulation. ~~Symbols represent individual healthy donors (*n* = 17).~~ *P* values were calculated with one-way repeated measures ANOVA, with Bonferroni correction for multiple comparisons. (**B**) Ratio of *IL13RA1-LOR1a*:*IL13RA1* expression in the same samples as in (A) treated with IL-13, stratified according to baseline *IL13RA1-LOR1a* expression. Each symbol represents purified primary B cells from an individual healthy human donor (*n* = 17).

Commented [SS5]: Align elements of figure left.

Commented [GK6R5]: We moved all elements in a single row.

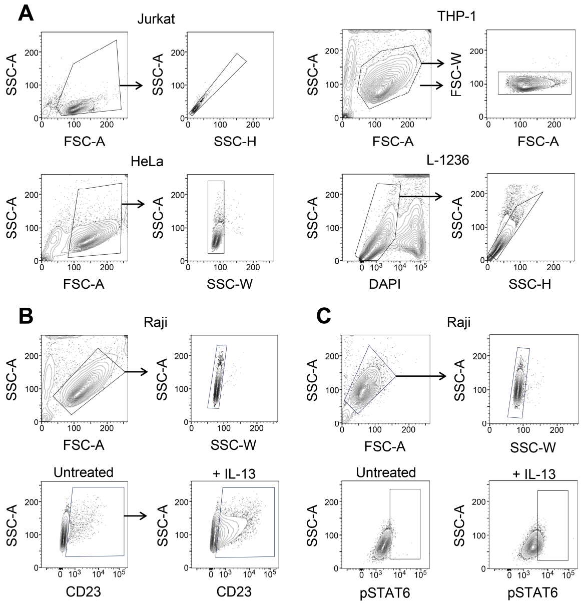

Fig. S18. Cell line flow cytometry gating strategies. (**A**) Representative flow cytometry plots displaying how indicated cell lines were gated. (**B**) Representative flow cytometry plots indicating how Raji cell lines were gated for CD23 expression with or without IL-13 treatment. (**C**) Representative flow cytometry plots indicating how Raji cell lines were gated for pSTAT6 expression with or without IL-13 treatment.

Table S1. Cell lines used in this study

Cell line	Species	Cell Origin	Media
HEK293T	Human	Embryonic kidney	IMDM + 5% FCS
HeLa	Human	Cervical adenocarcinoma	DMEM + 10% FCS
SUP-T1	Human	T lymphoblast	RPMI + 10% FCS
Jurkat	Human	T lymphoblast	RPMI + 10% FCS
HARA	Human	Lung squamous cell carcinoma	DMEM + 10% FCS
LK-2	Human	Lung squamous cell carcinoma	RPMI + 10% FCS + NEAA + 25 mM HEPES
HCC4006	Human	Lung adenocarcinoma	RPMI + 10% FCS
OE19	Human	Esophageal adenocarcinoma	DMEM + 10% FCS
PANC-1	Human	Pancreatic ductal adenocarcinoma	DMEM + 10% FCS
L-1236	Human	Hodgkin lymphoma	RPMI + 10% FCS
THP-1	Human	Monocytic leukaemia	RPMI + 10% FCS
Raji	Human	Burkitt lymphoma	RPMI + 10% FCS
U2OS	Human	Osteosarcoma	DMEM + 10% FCS
NCI-H358	Human	Lung adenocarcinoma	RPMI + 10% FCS
SQMK-FP	Squirrel monkey	Epithelial kidney	DMEM + 10% FCS
LLC-MK2	Rhesus monkey	Epithelial kidney	DMEM + 10% FCS
CP132	Chimpanzee	Skin fibroblast	DMEM + 10% FCS
CM0203F	Common marmoset	Bone marrow stromal cell	DMEM + 10% FCS

Table S2. PCR primers used in this study

Target	Forward primer (5′-3′)	Reverse primer (5′-3′)
HPRT1	TGACACTGGCAAAACAATGCA	GGTCCTTTTCACCAGCAAGCT
IL13RA1	TCCTGATCCTGGCAAGATTT	AGTCGGTTTCCTCCTTGGTT
IL13RA1-LOR1a	TCCTGATCCTGGCAAGATTT	TCAGATCCCGGATACTTTCTG
IL13RA1-AluJb	CCGCGGTGTGTCTACTGTGT	GCGACGATGACTGGAACA
IL13RA1 (end-point PCR)	CTACGGAAACTCAGCCAC	GCTCCAATTACTCCAGAG
IL13RA1-LOR1a (end-point PCR)	CTACGGAAACTCAGCCAC	GGAGTTGGTCAACAGGAAGC
IL13RA1-AluJb (end-point PCR)	CTACGGAAACTCAGCCAC	CATCAGTGTTTTAAATTTTAAT
IL13RA2	GTGGAGTGATAAACAATGCTGGG	TGGGTAGGTGTTTGGCTTACGC
IL4R	CACCTATGCAGTCAACATTTGGA	GATGCGGAGGGAGGGTTCTA
IL2RG	GTTCTCCTTGCCTAGTGTGGATGG	CCAACAGAGATAACCACGGCTTCC
JAK1	GAGACAGGTCTCCCACAAACAC	GTGGTAAGGACATCGCTTTTCCG
JAK2	CCAGATGGAAACTGTTCGCTCAG	GAGGTTGGTACATCAGAAACACC
TYK2	GGTTGACCAGAAGGAGATCACC	TCCTCGTCATCCATCTTGCCCT
STAT6	CCTTGGAGAACAGCATTCCTGG	GCACTTCTCCTCTGTGACAGAC
CCL26	GGGAGTGACATATCCAAGACCTG	CAGACTTTCTTGCCTCTTTTGGTA
IGHE (*86*)	ACTATGCCACCATCAGCTTG	GTTTTGTTGTCGACCCAGTC
S. boliviensis IL13RA1	TCCTGATCCTGGCAAGATTT	AGTCGGTTTCTTCCTTGGTT
C. jacchus IL13RA1	TGACACTGGCAAAACAATGCA	AGTCGGTTTCTTCCTTGATT
S. boliviensis, C. jacchus IL13RA1-LOR1a	TGACACTGGCAAAACAATGCA	TCAGCTCCTTGATACTATCTG
P. troglodytes, M. mulatta IL13RA1	TGACACTGGCAAAACAATGCA	AGTCGGTTTCCTCCTTGGTT
P. troglodytes IL13RA1-LOR1a	TGACACTGGCAAAACAATGCA	TCAGATCCTGGATACTTTCTG
M. mulatta IL13RA1-LOR1a	TGACACTGGCAAAACAATGCA	TCAGATCCTTGATACTATCTG

Table S3. ASOs, siRNA and sgRNA oligonucleotides used in this study

**Oligonucleotide**	**Sequence ~~5'~~5′-~~3'~~3′**
ASO_exon	CTTTCTGGGAGGCCTGATGG
ASO_splice	GGTTTGTTTCCTGAGAAAGG
siRNA_1 sense	GAUGGAAUUGAGACUGAUCUCUUGT
siRNA_1 antisense	ACAAGAGAUCAGUCUCAAUUCCAUCUC
siRNA_2 sense	GAGAUGGAAUUGAGACUGAUCUCTT
siRNA_2 antisense	AAGAGAUCAGUCUCAAUUCCAUCUCCC
siRNA_7 sense	GAAUUGAGACUGAUCUCUUGUCUCC
siRNA_7 antisense	GGAGACAAGAGAUCAGUCUCAAUUCCA
siRNA_9 sense	GUUUUGGUAACAUUACUACAGACCT
siRNA_9 antisense	AGGUCUGUAGUAAUGUUACCAAAACAG
sgRNA_1	GTTGCTGGCTTGAATACCAC
sgRNA_2	TCCGTAGACATATTAGAAGT
sgRNA_3	GGTTTGTCAGCCAATCAAGA
sgRNA_4	GATCCCTCACCCTTCTTGAT

Data file S1.
List of transcripts differentially expressed between LCLs with high and low IL13RA1 expression.

Data file S2.
Unmodified and uncropped versions of the blot images shown in the indicated figures.

Data file S3.
Tabulated data underlying Figs. 1 to 6 and figs. S1, S4 to S13, and S15 to S17.
